# Expression and functional analysis of the propamocarb-related gene *CsMAPEG* in cucumber

**DOI:** 10.1186/s12870-019-1971-z

**Published:** 2019-08-22

**Authors:** Fan Zhang, Zhiwei Qin, Xiuyan Zhou, Ming Xin, Shengnan Li, Jie Luan

**Affiliations:** College of Horticulture and Landscape Architecture, Key Laboratory of Biology and Genetic Improvement of Horticultural Crops (Northeast Region), Northeast Agricultural University, Harbin, 150030 China

**Keywords:** Cucumber, Cucumber downy mildew, Propamocarb, Functional analysis, *CsMAPEG*

## Abstract

**Background:**

Propamocarb (PM) is one of the main pesticides used for controlling cucumber downy mildew. However, due to its volatility and internal absorption, PM can easily form pesticide residues on cucumber fruits that seriously endanger human health and pollute the environment. The breeding of new cucumber varieties with a low abundance of PM residues via genetic methods constitutes an effective strategy for reducing pesticide residues and improving cucumber safety and quality. To help elucidate the molecular mechanism resulting in a low PM residue abundance in cucumber, we used the cucumber cultivar ‘D0351’ (which has the lowest PM residue content) as the test material and identified genes related to low PM residue abundance through high-throughput tag-sequencing (Tag-Seq).

**Results:**

*CsMAPEG* was constitutively expressed and showed both varietal and organizational differences. This gene was strongly expressed in ‘D0351’. The expression levels of *CsMAPEG* in different cucumber tissues under PM stress were as follows: fruit>leaf>stem>root. *CsMAPEG* can respond to salicylic acid (SA), gibberellin (GA) and *Corynespora cassiicola* Wei (Cor) stress and thus plays an important regulatory role in plant responses to abiotic and biological stresses. The PM residue abundance in the fruits of *CsMAPEG*-overexpressing plants was lower than those found in antisense *CsMAPEG* plants and wild-type plants at all tested time points. The results revealed that *CsMAPEG* played a positive role in reducing the PM residue abundance. A *CsMAPEG* sense construct increased the contents of SOD, POD and GST in cucumber fruits, enhanced the degradation and metabolism of PM in cucumber, and thus effectively reduced the pesticide residue abundance in cucumber fruits.

**Conclusions:**

The expression patterns of *CsMAPEG* in cucumber cultivars with high and low pesticide residue abundances and a transgenic verification analysis showed that *CsMAPEG* can actively respond to PM stress and effectively reduce the PM residue abundance in cucumber fruits. The results of this study will help researchers further elucidate the mechanism responsible for a low PM residue abundance in cucumber and lay a foundation for the breeding of new agricultural cucumber varieties with low pesticide residue abundances.

**Electronic supplementary material:**

The online version of this article (10.1186/s12870-019-1971-z) contains supplementary material, which is available to authorized users.

## Background

Cucumber is a staple vegetable in China that is cultivated throughout the country, primarily through protected cultivation. Because of the special microclimate in the protected cultivation environment, continuous cropping and other factors, downy mildew is commonly observed during cucumber production. Cucumber downy mildew can be found in plants from the seedling to adult stages, and severe cases can cause yield losses of 20–40% [1]. Therefore, high levels of propamocarb (PM) are used to control this disease. Due to the lack of suitable substitutes for controlling cucumber downy mildew, nearly 80% of farmers in China use PM to control cucumber downy mildew [2]. PM is a low-toxicity fungicide that can effectively control cucumber downy mildew. However, due to its volatility and internal absorption, PM can pollute the environment and easily form pesticide residues on cucumber fruit [3], and these pesticide residues on cucumber are regarded as neurotoxicants that seriously endanger human health. Animal experiments have confirmed that pesticide residues exert carcinogenic effects [4–7], and a pathogenic analysis has revealed that the fungicide PM (carbamate) can inhibit the decomposition of acetylcholinesterase to induce the accumulation of acetylcholine in the body, which would affect the normal nerve conduction process in organisms and even lead to the poisoning and death of organisms [8]. Therefore, PM residues on crops have attracted increasing attention due to food safety and health concerns. A system for the evaluation of pesticide residues in cucumber germplasm resources has been established, and the botanical morphological characteristics have been clarified. Germplasm resources with low and high residue abundances of the pesticides deltamethrin, PM and myclobutanil have been identified [9]. Previous studies have performed genetic analyses of cucumber PM residue, have revealed that the abundance of PM residues in cucumber fruits is a quantitative trait controlled by multiple genes, and have detected a QTL related to PM residue abundance [10]. The fluidity and changes in the PM residue abundance in plants have also been analyzed. Varieties with a low pesticide residue abundance exhibit a lower residue quantity and a decreased rate of change in the residue quantity compared with varieties with a high pesticide residue abundance [11]. Based on the results from physiological and biochemical studies of cucumbers with low PM residue abundances, the differentially expressed genes in cucumber fruits under PM stress have been screened through Solexa and comparative genomics methods. The identified genes can be divided into four main categories: (1) transcription factors involved in transcriptional regulation, such as WRKY and DREB; (2) pesticide detoxification-related genes, such as the ABC family, cytochrome P450 family, and GSTs; (3) signal transduction-related genes, such as aminotransferase and succinate dehydrogenase; and (4) stress- and stimulation-related genes, such as heat shock proteins (HSPs) and cysteine synthase (CYS) [12]. Studies of the *CsABC19* [13], *CsWRKY30* [14], *CsSDH* [15] and *CsDIR16* [16] genes, which respond to PM stress in variants with high and low PM residue abundances, have shown that these differentially expressed genes can significantly improve the resistance of transgenic *Arabidopsis thaliana* or cucumber to PM stress.

Glutathione (GSH) is involved in biological transformations and promotes the transformation of harmful toxins in the body into harmless substances that are then excreted. GSH can directly participate in metabolic reactions as a substrate. This molecule also induces the expression of detoxification genes, activates detoxification enzymes, such as GST, in plants, promotes detoxification and metabolic reactions, and thus effectively reduces the abundance of pesticide residues in plants [17, 18]. In vivo studies have revealed that GSH-related pathways play an important role in pesticide degradation and metabolism in maize, wheat and tomato plants [19–21]. Reactive oxygen species (ROS) can be produced by plants under stress, and their production can lead to the peroxidation of membrane lipids to malondialdehyde (MDA). After MDA enters cells, it can react with proteins and nucleic acids and thereby induce losses in the functions of these compounds. Therefore, the MDA content can reflect the degree of damage in plants under stress. However, GST can eliminate ROS to maintain normal life metabolism activities in plants [22–24]. Protective enzymes (mainly SOD and POD) are found in the membrane system of cucumber. SOD can catalyze the disproportionation of O_2_ to H_2_O_2_, and POD can reduce H_2_O_2_ to H_2_O, can efficiently scavenge oxygen free radicals and thus increase the metabolic capacity of plants for toxic substances [25]. The metabolic membrane-related proteins that form part of MAPEG family include GSH and arachidonic acid. Most members of the MAPEG family, which are related to cell detoxification and drug resistance, are involved in the synthesis of the inflammatory mediators LTs and PGE2 and in the promotion of in vivo anti-electron affinity, oxygen free radical attack, and exogenous lipid metabolism. Studies have shown that MAPEG proteins regulate liver injury in mice and beagles and exert detoxification effects [26, 27]. In recent years, toxicology and pharmacology research on the functions of the MAPEG family has rapidly progressed from the known involvement of this family in arachidonic acid and GSH metabolism, but the detoxification mechanism of the MAPEG family proteins in plants remains unclear.

Thus far, many genes related to drug detoxification have been identified. To study the role of these detoxification-related genes in the detoxification of fungicides in cucumber, Solexa high-throughput tag sequencing was performed [12], and the results were used to identify the candidate gene Cucsa.034680.1 involved in the GSH pathway. This gene, which is named *CsMAPEG*, belongs to the MAPEG protein family. The results reported by Wu Peng showed that the activity of glutathione transferase is significantly increased after treatment with PM [2]. A fluorescence quantitative analysis previously showed that *CsMAPEG* is upregulated in the low-pesticide-residue-abundance cucumber cultivar `D0351’ under PM stress and could exhibit a significant response to PM threat. However, the mechanism underlying the response of *CsMAPEG* to PM stress has not been reported. The cloning of *CsMAPEG* using ‘D0351’ fruit cDNA as the template and a bioinformatics analysis of the sequence domains and gene structure were performed to explore the physicochemical properties of this protein as well as its homologous sequences and subcellular localization. The expression of *CsMAPEG* in strains with high and low abundances of PM, the residual quantity of PM in *CsMAPEG*-overexpressing plants and the related physiological and biochemical indexes were analyzed. The aims of this study were to explore the performance of *CsMAPEG* under PM stress and to help elucidate the mechanism underlying the production of low abundances of PM residues in cucumber, and the results will contribute to the breeding of new cucumber varieties with a low abundance of pesticide residues.

## Results

### Cloning and bioinformatics analysis of *CsMAPEG*

**Fig. 1:**
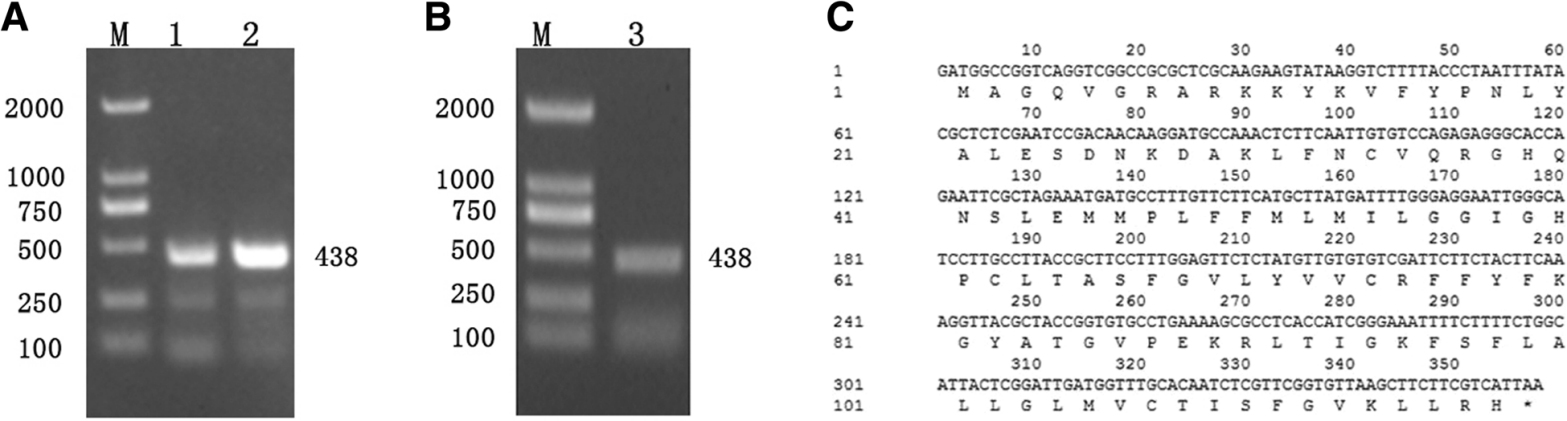
Cloning of CDS sequences of *CsMAPEG* and Sequence analysis. **a**:Gel electrophoresis of PCR products. Lane M: 2000 bp DNA Marker (Toyobo, Japan); Lane 1, 2:PCR amplification of *CsMAPEG*. **b**:PCR of *E. coli* DH5α transformed *CsMAPEG.* Lane M: 2000 bp DNA Marker (Toyobo, Japan); Lane 3:PCR amplification of *CsMAPEG*. **c**:The cDNA sequence and corresponding amino acid suquence of *CsMAPEG*

*CsMAPEG* was amplified by PCR using *CsMAPEG*-F and *CsMAPEG*-R as the primers and cucumber ‘D0351’ fruit cDNA as the template (Fig. 1a). The *CsMAPEG* sequence was then confirmed by PCR and repeated sequencing (Fig. 1b). Full-length *CsMAPEG* is 438 bp in length and encodes 145 amino acids (Fig. 1c). Our sequence was consistent with the coding region of the gene (Csa5G409710.1) obtained from the alignment of sequences in the cucumber genome database (http://www.icugi.org/).

The amino acid sequence alignment of *CsMAPEG* showed that the protein has one MAPEG domain (Additional file 3: Figure S1) and belongs to the MAPEG superfamily; thus, the protein was named *CsMAPEG*. The analysis of *CsMAPEG* revealed a predicted theoretical molecular weight of 16.486 kDa, a theoretical isoelectric point of 9.66, an atomic composition of C_775_H_1193_N_189_O_186_S_11_, a total number of atoms of 2354, and a fat coefficient of 104.83. The highest hydrophobicity scores of the protein encoded by this gene (Additional file 4: Figure S2) were found to equal 2.944 at the 16th amino acid position and 2.122 at the 67th amino acid position, and the average hydrophilic coefficient was estimated as − 0.538, which indicated that the protein is hydrophilic. A clear signal peptide cleavage site was not detected for this protein (Additional file 5: Figure S3), which suggested that *CsMAPEG* might be a cytoplasmic matrix or organelle matrix protein but not a membrane or secretory protein. The prediction of the transmembrane domain of the *CsMAPEG* protein showed three distinct transmembrane regions (Additional file 6: Figure S4). The secondary structure of the protein (Additional file 7: Figure S5) was analyzed, and the analysis revealed 83 alpha helices, which accounted for 57.24% of the total polypeptide chain, 20 extended main chains, which accounted for 13.79% of the whole polypeptide chain, and 36 random coils, which accounted for 24.83% of the whole polypeptide chain. The protein sequence was submitted to the protein homology modeling program Phyre to predict the three-level structure of the protein (Additional file 8: Figure S6).

The cucumber genome database was searched for Csa5G409710.1 to obtain the complete gene sequence, and a region 2000 bp upstream was selected as the promoter sequence. The analysis results are shown in Additional file 1: Table S1. In addition to the TATA and CAAT elements inherent in the eukaryotic promoter and some common light-responsive elements, such as the 3-AF1-binding site, GT1 motif and SP1-binding site, the promoter also contains an ARE anaerobic induction element, a P-box gibberellin (GA) response element, an ERE ethylene response element, a TC-rich repeat defense and stress response element, three heat shock reaction elements, one TCA-element salicylic acid (SA) response element, one AuxRR-core auxin response element and other cis-elements.

### Phylogenetic tree of *CsMAPEG*

The amino acid sequences of MAPEG family proteins from 10 different specieswere downloaded from the NCBI database (https://www.ncbi.nlm.nih.gov/) for homology alignment. A phylogenetic tree was constructed using the neighbor-joining (NJ) method with MEGA software (version 5.2). The results are shown in Additional file 9: Figure S7, and the scale bar indicates the average number of amino acid substitutions per site. Fifteen family members (Additional file 2: Table S2), including *CsMAPEG*, were grouped into two groups. The MAPEG protein shows strong conservation among different species, and the proteins from plants in the same family and genus show high similarity. *CsMAPEG* and the protein from melon *Cucumis melo* L. (XM008462283.2) form a small evolutionary branch and show the closest relationship; in addition, these proteins exhibit a distance of less than 0.1 from the proteins of balsam pear *Momordica charantia* L. (XM022285675.1) and pumpkin *Cucurbita moschata* (XM023137096.1), which belong to Cucurbitaceae. The change in nucleotides among these proteins is small, and their homology is relatively high.

### Subcellular localization of the *CsMAPEG* protein

**Fig. 2:**
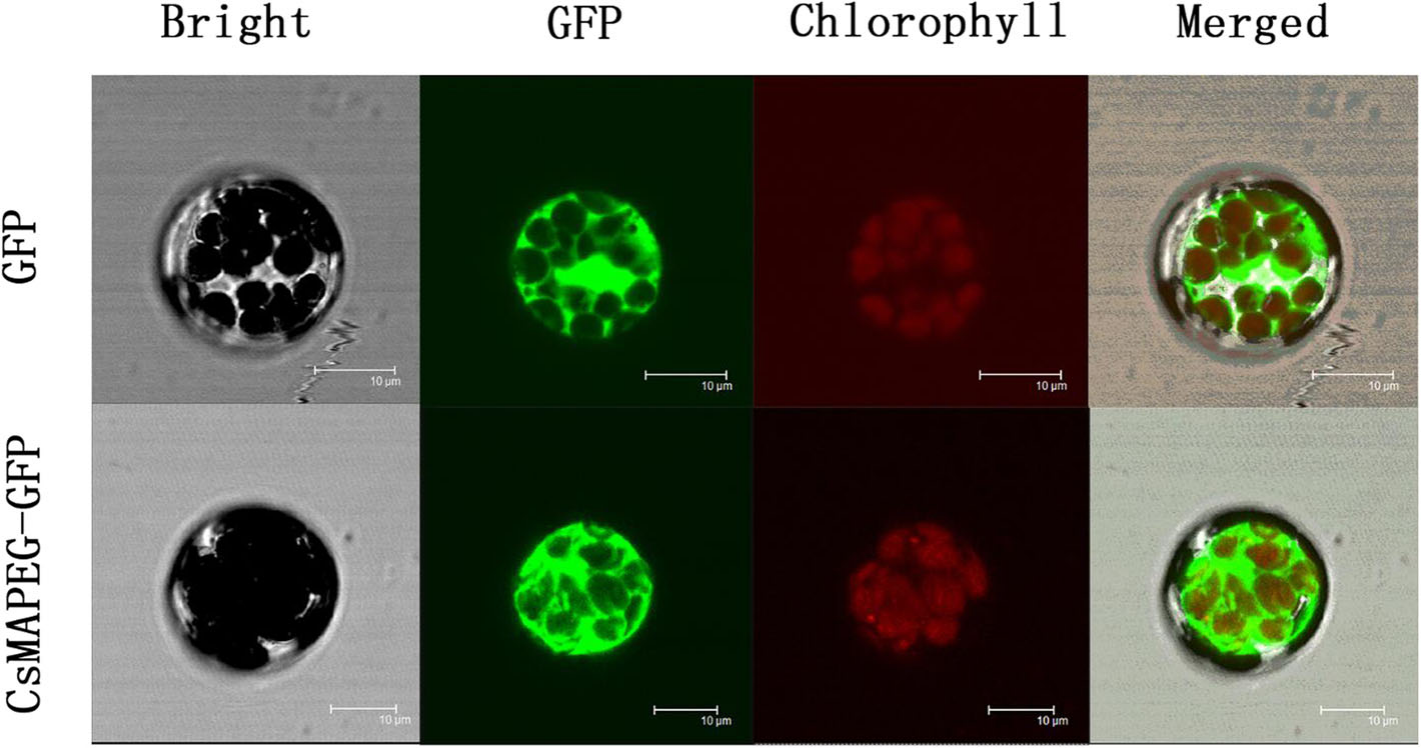
Subcellular localization of CsMAPEG-pGII-EGFP protein in Arabidopsis protoplasts. Subcellular localization of the CsMAPEG-pGII-EGFP fusion protein in Arabidopsis protoplasts. Images show protoplasts prepared from 3- to4-week-old Arabidopsis leaves expressing CsMAPEG-pGII-EGFP (bottom row) or pGII-EGFP (upper row). Bright-field illumination, GFP fluorescence, chlorophyll fluorescence, and an overlay of GFP and chlorophyll fluorescence are shown. Scale bars, 10 μm

The 35S:*CsMAPEG*-GFP fusion expression vector and the pGII-EGFP empty vector were introduced into *Arabidopsis* protoplasts. The subcellular localization of the *CsMAPEG* fusion protein was observed using a confocal laser microscope, and the results are shown in Fig. 2. Bright green fluorescence was observed in whole cells transfected with the empty vectors, whereas enriched green fluorescence following transfection with the *CsMAPEG*-pGII-EGFP fusion expression vector was observed only in the cytoplasm. Therefore, the results clearly indicated that *CsMAPEG* is localized in the cytoplasm.

### Expression pattern of *CsMAPEG* in response to PM treatment

**Fig. 3:**
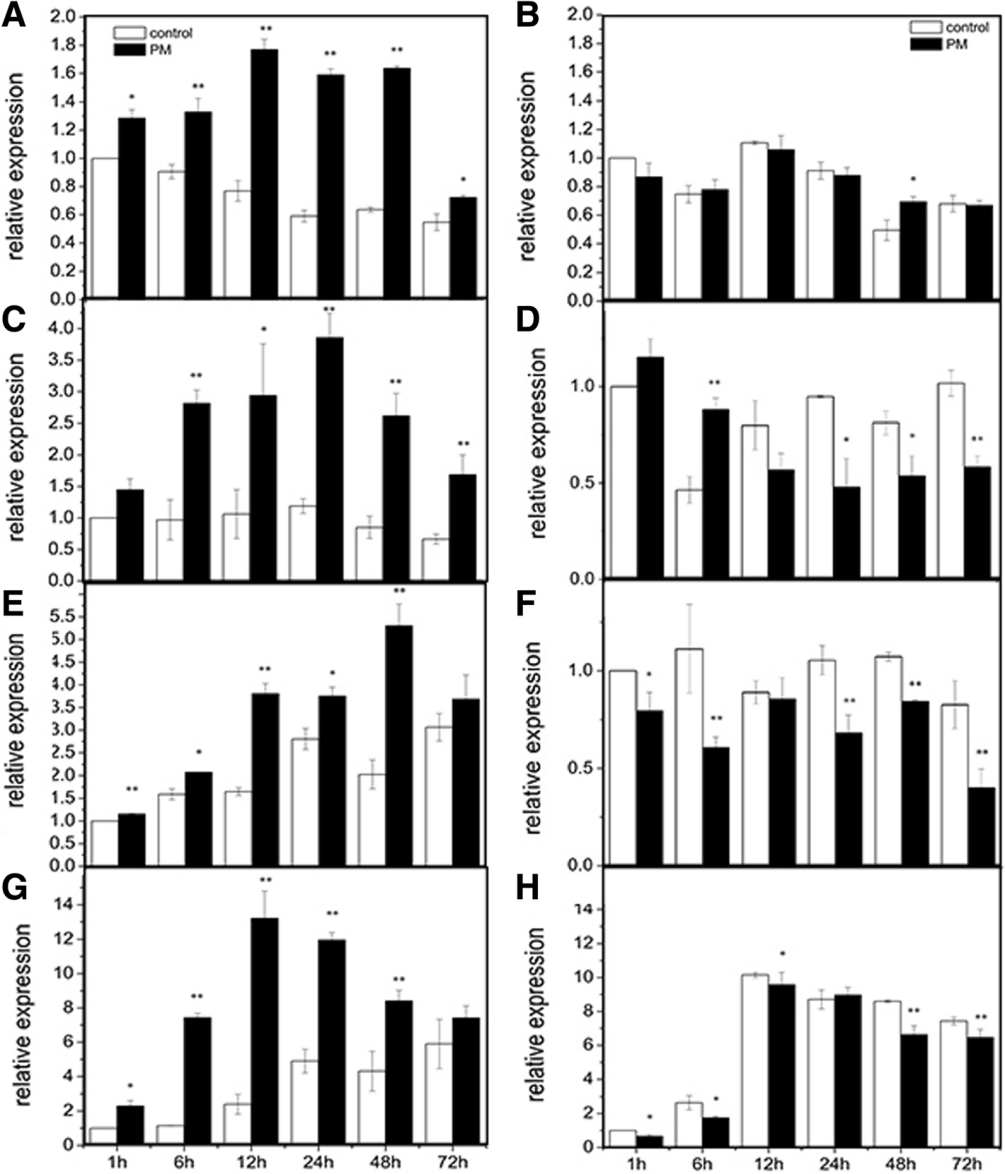
Expression pattern of *CsMAPEG* in response to propamocarb stress. **a**: roots of D0351; **b**:roots of D9320; **c**:stem of D0351; **d**:stem of D9320; **e**:leaf of D0351; **f**:leaf of D9320; **g**:fruit of D0351; **h**:fruit of D9320; the “*” presents the value is extremely significant at 0.05 level based on student t test; the “**” presents the value is extremely significant at 0.01 level based on student *t* test

The expression patterns of *CsMAPEG* in cucumber plants treated with PM showed differences between the low-PM-residue-abundance cultivar ‘D0351’ and the high-PM-residue-abundance cultivar ‘D9320’ (Fig. 3). Exposure to PM increased the expression levels of *CsMAPEG* in the roots, stems, leaves and fruits of ‘D0351’ over time compared with the expression levels found in the same tissues of ‘D9320’. At all the tested time points, significantly higher *CsMAPEG* expression was found in ‘D0351’ roots compared with the control group, and specifically, the expression level in the ‘D0351’ roots at 24 h was 2.69-fold higher than that the control level (Fig. 3a). However, *CsMAPEG* expression in ‘D9320’ was significantly downregulated at 48 h, and the expression levels at the other time points were not significantly different from those of the control group (Fig. 3b). The expression levels of *CsMAPEG* in the stems of ‘D0351’ were first upregulated and then downregulated. The relative expression of *CsMAPEG* in the ‘D0351’ stems at each time point was significantly higher than that in the control group and reached its maximal level, which was 3.24-fold higher than that of the control group, at 24 h (Fig. 3c). The levels in ‘D9320’ were decreased significantly compared with those of the control at all the tested time points with the exception of 1 and 6 h, and the overall expression level showed a smooth change and was significantly lower than the expression level in ‘D0351’ (Fig. 3d). The expression of *CsMAPEG* in the leaves of ‘D0351’ was upregulated at all tested time points compared with that of the control. The relative expression level reached its maximum at 48 h, and this maximal level was 2.61-fold higher than the control level (Fig. 3e). However, *CsMAPEG* expression in ‘D9320’ leaves was downregulated at all time points; in addition, the change in *CsMAPEG* expression in ‘D9320’ leaves was stable, and the levels were significantly lower than those in ‘D0351’ (Fig. 3f). The patterns of *CsMAPEG* expression in ‘D0351’ and ‘D9320’ fruits were basically consistent, characterized by an initial increase and a subsequent decrease, although the gene expression level in ‘D0351’ at all tested time points was significantly higher than that in ‘D9320’. The relative expression of *CsMAPEG* in ‘D0351’ fruits increased rapidly within 6 h and reached its maximum at 12 h, and this maximal expression level was 5.49-fold higher than that of the control group. After 12 h, the expression level decreased, although the values remained higher than those of the control at all time points (Fig. 3g). The *CsMAPEG* expression levels in ‘D9320’ fruits were upregulated only at 6 and 24 h, and these values were 1.81- and 1.34-fold higher than those of the control group, respectively. The relative expression of *CsMAPEG* at the other time points was lower than that of the control group (Fig. 3h). The *CsMAPEG* expression levels in different organs of the low-pesticide-residue-abundance cultivar ‘D0351’ were ordered as follows: fruit>leaf>stem>root. The expression of *CsMAPEG* in the stems and leaves of ‘D0351’ was significantly higher than that in the same parts of ‘D9320’. *CsMAPEG* was specifically expressed in the stems and leaves of ‘D0351’ compared with those of ‘D9320’.

### Analysis of *CsMAPEG* expression in response to different external factors

**Fig. 4:**
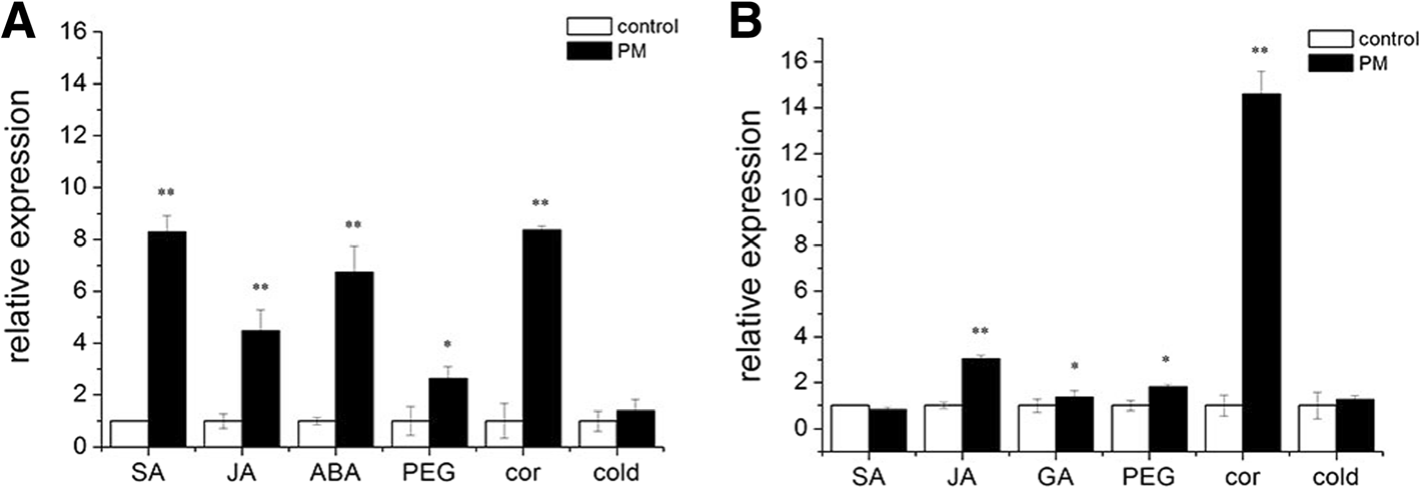
Expression pattern of *CsMAPEG* under different stress of ‘D0351’ and ‘D9320’. **a**:Cucumber variety ‘D0351’; **b**:Cucumber variety ‘D9320’.the “*” presents the value is extremely significant at 0.05 level based on student t test; the “**” presents the value is extremely significant at 0.01 level based on student *t* test

The expression patterns of *CsMAPEG* after ‘D0351’ and ‘D9320’ seedlings at the three-true-leaf stage were treated with the hormones jasmonic acid (JA), SA and GA were analyzed. The results (Fig. 4a and b) showed that SA induction significantly upregulated the expression of *CsMAPEG* in the low-PM-residue-abundance cultivar ‘D0351’ to a level that was 8.76-fold higher than that of the control. The expression of *CsMAPEG* in the high-PM residue-abundance cultivar ‘D9320’ was not significantly different from that of the control. The JA-induced expression patterns of *CsMAPEG* in ‘D0351’ and ‘D9320’ were similar and showed very significant upregulation, although the relative expression of *CsMAPEG* in ‘D0351’ was higher than that in ‘D9320’. The expression of *CsMAPEG* in ‘D0351’was 4.47-fold higher than that of the control and 3.03-fold higher than that of the ‘D9320’ cultivar. After GA treatment, *CsMAPEG* showed different expression patterns in ‘D0351’ and ‘D9320’. Its expression was upregulated significantly in ‘D0351’ to a value that was 6.73-fold higher than that in the control, and this value was only slightly higher than that in ‘D9320’, which presented a relative expression level that was 1.36-fold higher than that in the control. In ‘D0351’, *CsMAPEG* expression was upregulated after SA, JA and GA induction. In contrast, in ‘D9320’, *CsMAPEG* expression was upregulated only after JA treatment and did not show significant changes after the SA and GA treatments. However, a significant difference in the *CsMAPEG* expression pattern was found between ‘D0351’ and ‘D9320’. To study the function of the MAPEG family in stress tolerance, we subjected seedlings of ‘D0351’ and ‘D9320’ at the three-true-leaf stage to drought, Cor and low temperature stress and analyzed the resulting expression patterns of *CsMAPEG*. As shown in Fig. 4, *CsMAPEG* expression in ‘D0351’ and ‘D9320’ under PEG stress was upregulated, and these upregulated levels were significantly different from that of the control. The relative expression of *CsMAPEG* in ‘D0351’ was significantly higher than that in ‘D9320’, and the levels in these strains were 2.63- and 1.83-fold higher than that in the control, respectively. The expression of *CsMAPEG* was significantly upregulated in ‘D0351’ and ‘D9320’ under Cor stress. The expression of *CsMAPEG* in these strains was 14.60- and 8.36-fold higher than that in the control, respectively. Under low temperature stress, the expression of *CsMAPEG* in ‘D0351’ and ‘D9320’ was nearly equivalent, and no significant difference compared with the control was detected.

### Construction of the *CsMAPEG* expression vector and genetic transformation of cucumber

**Fig. 5:**
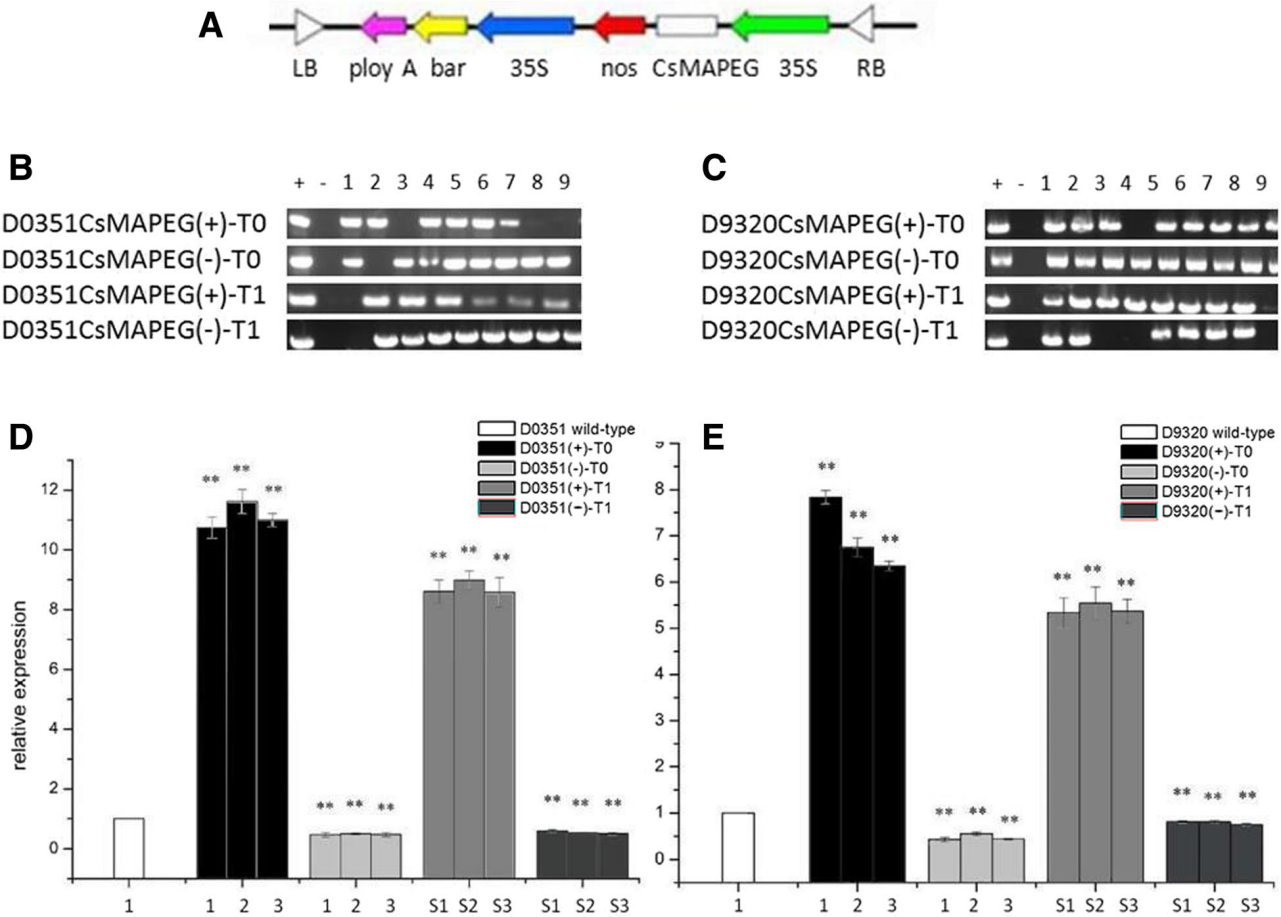
Molecular biological verification of transgenic plants. **a**: Construction of plant vector pCXSN-CsMAPEG;**b**: ‘D0351’transgenic plants T_0_ and T_1_ were identified by PCR; **c**: ‘D9320’transgenic plants T_0_ and T_1_ were identified by PCR;**d**: Relative transcript levels of CsMAPEG in ‘D0351’transgenic plants T_0_ and T_1_;**e**: Relative transcript levels of CsMAPEG in ‘D9320’ transgenic plants T_0_ and T_1_

We generated the overexpression vectors *CsMAPEG*(+)-PCXSN and *CsMAPEG*(−)-PCXSN under the control of the strong constitutive CaMV35S promoter (Fig. 5a). The overexpression vectors *CsMAPEG*(+)-PCXSN and *CsMAPEG*(−)-PCXSN were successfully transferred into ‘D0351’ and ‘D9320’ using cucumber genetic transformation technology (Additional file 10: Figure S8). The total DNA of the resistant plants was used as a template, the pCXSN-*CsMAPEG*(+) plasmid was used as a positive control, and water was used as a negative control. The primers used for PCR detection were specific for the pCXSN-1250 vector. As shown in Fig. 5b and c, the target fragments were approximately 500 bp in the positive control and some resistant plants, although no specific bands were found in the negative control, which indicated that the pCXSN-*CsMAPEG* plasmid had been successfully integrated into the cucumber genome. To eliminate false positives in the resistant plants and ensure the integrity and accuracy of the experiment, we extracted RNA using the TRIzol method and identified the sequence by qRT-PCR. The normal expression of *CsMAPEG* in cucumber was affected by the transfection of exogenous genes, and the expression level was changed. The expression patterns were significantly different. The three *CsMAPEG*-overexpressing plants with the highest levels of *CsMAPEG* were selected for the detection of PM residues and the determination of physiological and biochemical indexes. In ‘D0351’, the expression of *CsMAPEG* was significantly upregulated after the transfer of *CsMAPEG*(+), and the *CsMAPEG* expression level in the T0 and T1 plants was 11.12- and 8.72-fold higher than that in the wild-type, respectively. After the transfer of *CsMAPEG*(−), the expression of *CsMAPEG* in the T0 and T1 plants was downregulated to levels that were 0.48- and 0.55-fold of the wild-type level (Fig. 5d). In ‘D9320’, the expression of *CsMAPEG* was upregulated after the transfer of *CsMAPEG*(+), and the expression levels in the T0 and T1 plants were approximately 6.98- and 5.41-fold higher than that of the wild-type. After the transfer of *CsMAPEG*(−), the expression levels of *CsMAPEG* in the T0 and T1 plants were approximately 0.48- and 0.79-fold of the wild-type level (Fig. 5e).

### Analysis of PM residues in *CsMAPEG*-overexpressing plants

**Fig. 6:**
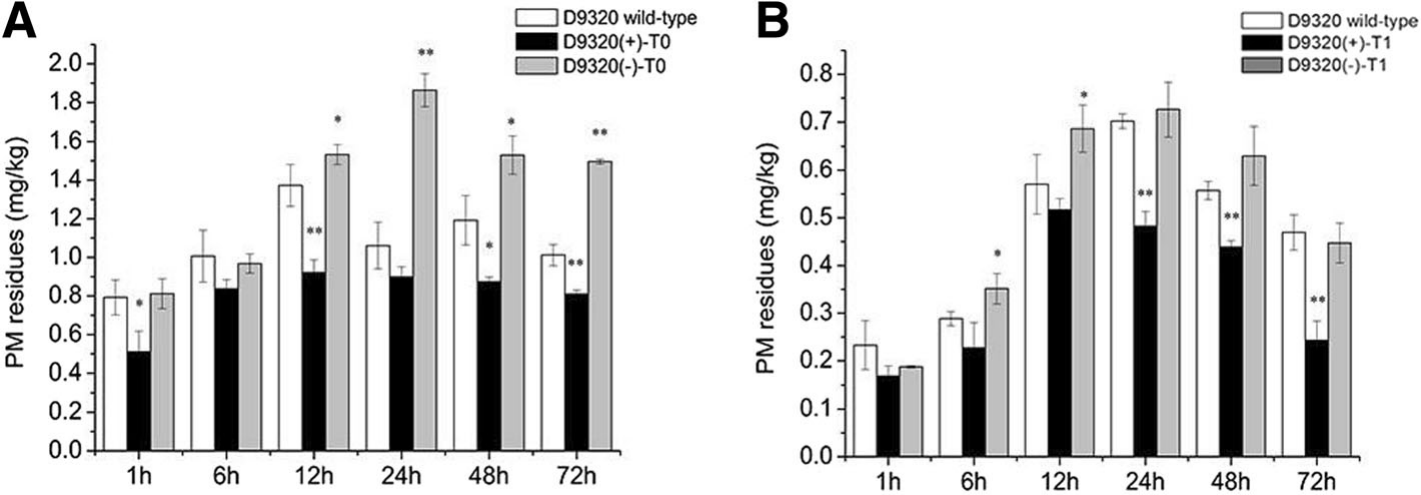
*CsMAPEG* residue of ‘D9320’ transgenic plant. **a**: Quantitative analysis of PM residues in ‘D9320’ T_0_ transgenic plants using gas chromatography; **b**: Quantitative analysis of PM residues in ‘D9320’ T_1_ transgenic plants using gas chromatography. The “*” presents the value is extremely significant at 0.05 level based on student t test; the “**” presents the value is extremely significant at 0.01 level based on student *t* test

‘D9320’ plants with similar expression levels of *CsMAPEG* were identified by PCR and qRT-PCR, and the PM residues in fruits after treatment with PM were determined (Fig. 6). The levels of residues in *CsMAPEG*(+)-overexpressing cucumber fruits at six time points from 1 to 72 h were significantly lower in the ‘D9320’ T0-generation fruits compared with the wild-type control fruits, and the effect was extremely significant at 12 and 72 h, with levels that were 0.67- and 0.80-fold of those found in the wild-type fruits, respectively. The average from the six time points obtained from the ‘D9320’ T0-generation fruits was 0.81 mg/kg, which was 0.26 mg/kg lower than the average value found for the wild-type fruits. The results showed that the *CsMAPEG*(+) transfer could effectively reduce the PM residues in fruits. The residue abundance in the *CsMAPEG*(−) transgenic plants at four time points from 12 h to 72 h was significantly or extremely significantly higher than that in the wild-type plants. The highest residue level of 1.86 mg/kg was found at 24 h. The residue abundances in the *CSMAPEG*(−) plants at 24 and 72 h were extremely significantly higher than those of the wild-type, and these values were 1.76- and 1.48-fold higher than those in the wild-type, respectively. The average residue abundance in the *CsMAPEG*(−) transgenic cucumber fruits at six time points was 1.37 mg/kg, which was 0.29 mg/kg higher than that found for the wild-type plants.

The PM residue abundance in the *CsMAPEG*(+)-overexpressing, *CsMAPEG*(−)-overexpressing and wild-type plants at the T1 generation showed the same trend; specifically, the PM residue abundance first increased and then decreased over time. Compared with the T0 plants, these T1 plants showed decreased PM residue abundances at all tested time points. The PM residue abundances in the *CsMAPEG*(+)-overexpressing plants were lower than those in the wild-type plants at the six tested time points. Specifically, the residue levels in the *CsMAPEG*(+)-overexpressing plants were 0.69-, 0.79- and 0.52-fold of those of the control plants at 24, 48 and 72 h, respectively, and these differences were significant. The average value from six time points was 0.35 mg/kg, which was 0.12 mg/kg lower than the average value obtained for the wild-type. The PM residue abundance in the *CsMAPEG*(−)-overexpressing T1 plants first increased significantly, reached a maximum of 0.73 mg/kg at 24 h, and then decreased slowly. The PM residues in these plants were higher than those in the wild-type at all time points with the exception of 1 and 72 h, when slightly lower residue abundances were detected in these plants relative to the wild-type plants. The average residue abundance of the *CsMAPEG*(−) transgenic cucumber fruit was 0.50 mg/kg, which was 0.03 mg/kg higher than that in the wild-type plants.

**Fig. 7:**
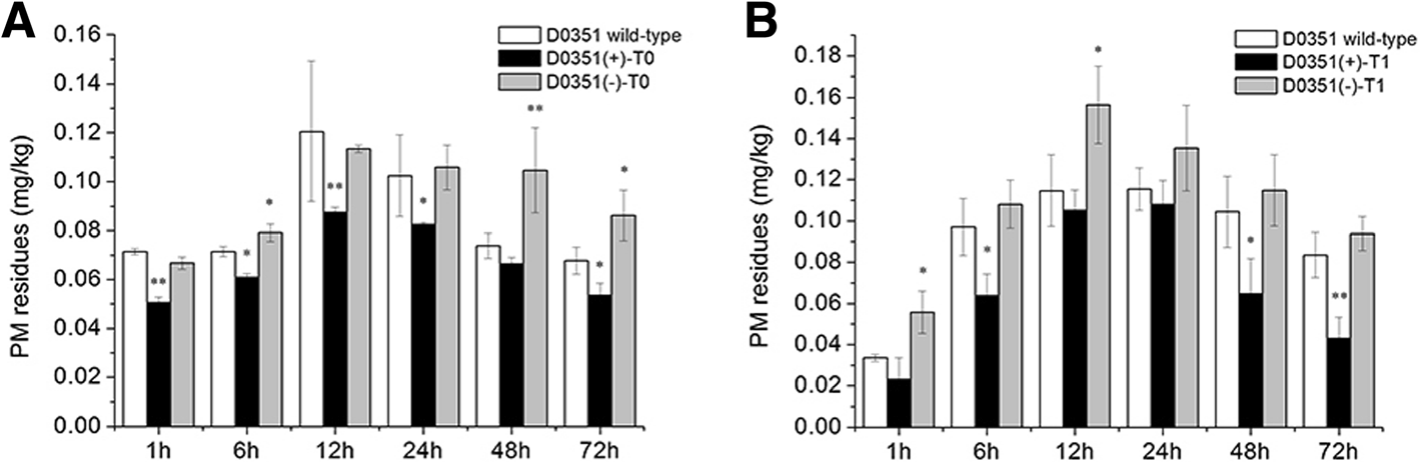
*CsMAPEG* residue of ‘D0351’ transgenic plant. **a**: Quantitative analysis of PM residues in ‘D0351’ T_0_ transgenic plants using gas chromatography; **b**: Quantitative analysis of PM residues in ‘D0351’ T_1_ transgenic plants using gas chromatography. The “*” presents the value is extremely significant at 0.05 level based on student *t* test; the “**” presents the value is extremely significant at 0.01 level based on student *t* test

‘D0351’ plants with similar expression levels of *CsMAPEG* were identified by PCR and qRT-PCR, and the PM residues in fruits after treatment with PM were determined (Fig. 7). The residue abundances in *CsMAPEG*(+)-overexpressing cucumber fruits at six time points from 1 to 72 h were lower in the ‘D0351’ T0-generation fruits than in the wild-type fruits. The maximum residue abundance of 0.09 mg/kg was observed at 12 h. The residue abundances at 6, 24 and 72 h were 0.85-, 0.80- and 0.78-fold of those of the wild-type plants, respectively, and these differences were significant. In addition, the residue abundances in the *CsMAPEG*(+) plants at 1 and 12 h were extremely significantly lower than those in the wild-type plants, and the values obtained for the *CsMAPEG*(+) plants were 0.71- and 0.67-fold of the wild-type values, respectively. The average value from six time points was 0.07 mg/kg, which was 0.02 mg/kg lower than the average value obtained for the wild-type. The results showed that *CsMAPEG*(+) transfer could significantly reduce the PM residue abundance in cucumber fruits. The residue abundances in the *CsMAPEG*(−)-overexpressing cucumber fruits were significantly higher than those in the wild-type plants at six time points from 1 to 72 h, with the exception of 1 and 12 h, when slightly lower values were detected. The average residue abundance in the *CsMAPEG*(−)-overexpressing plants was 0.09 mg/kg, which was 0.01 mg/kg higher than that obtained for the wild-type. The residue abundances in these plants at 6 and 72 h were 1.11- and 1.28-fold higher compared with the wild-type levels, respectively, and these differences were significant. In addition, the residue abundance in these plants at 48 h was 1.43-fold higher than that in the wild-type plants, and this difference was extremely significant.

The trend found for the variation in the PM residue abundance in CsMAPEG T1-generation transgenic plants was consistent with that found in the T0-generation plants and was characterized by an initial increase followed by a decrease. The residue abundances in the *CsMAPEG*(+)-overexpressing plants were lower than those in the wild-type plants at the six tested time points. After reaching the maximal value of 0.11 mg/kg at 24 h, the residue abundance showed a significantly decreasing trend. The values found for the *CsMAPEG*(+)-overexpressing plants at 6 and 48 h were 0.66- and 0.62-fold of the wild-type values, respectively, and these differences were significant; in addition, an extremely significant difference, which corresponded to a 0.52-fold change, was found at 72 h. The average PM residue abundance in CsMAPEG(+)-overexpressing T1-generation plants was 0.07 mg/kg, which was lower than that found for the wild-type plants (0.02 mg/kg). The residue abundance in the CsMAPEG(−)-overexpressing plants was higher than that in the wild-type plants at all tested time points, and significant 1.66- and 1.36-fold changes were detected at 1 and 12 h, respectively. The average residue abundance in the *CsMAPEG*(−)-overexpressing plants was 0.11 mg/kg, which was 0.02 mg/kg higher than that found for the wild-type plants.

### Physiological and biochemical indexes under PM treatment

#### POD analysis of *CsMAPEG*-overexpressing plants

**Fig. 8:**
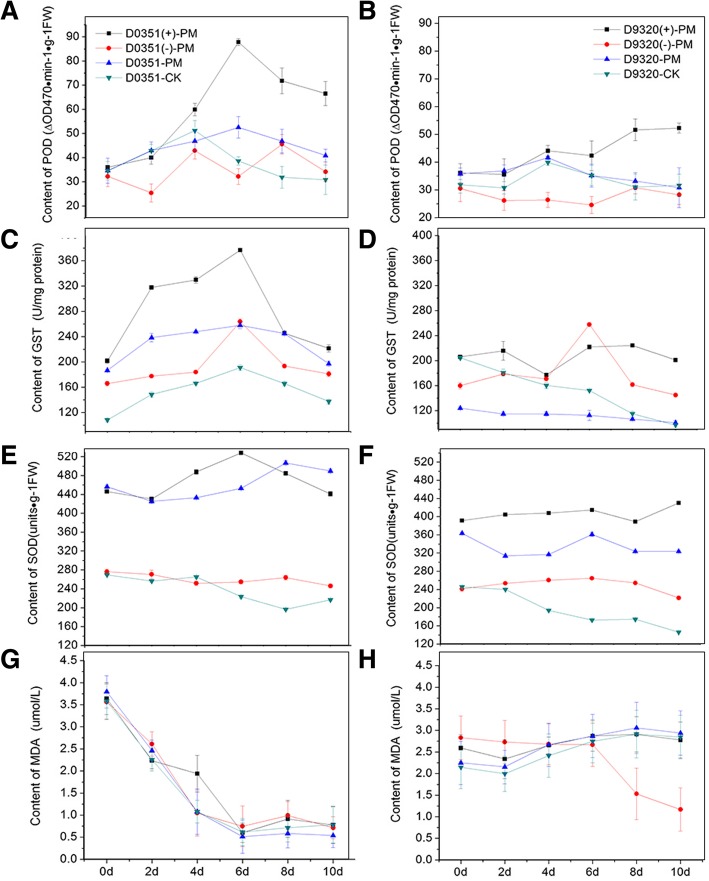
Analysis of physiological indexes under T_0_ stress of PM. **a**: ‘0351’ POD content; **b**: ‘9320’ POD content; **c**: ‘0351’ GST content; **d**: ‘9320’ GST content; **e**:‘0351’ SOD content; **f**: ‘9320’ SOD content;**g**: ‘0351’ MDA content;**h**: ‘9320’ MDA content

As shown in Fig. 8a and b, most treatments of T_0_ plants resulted in a trend for enzyme activity consisting of an increase followed by a decrease, and the POD enzyme activity reached a maximal level after 6 days. A comparison of the ‘D0351’ and ‘D9320’ wild-type plants treated with distilled water showed that the enzyme activity in ‘D0351’ plants at each time point was higher than that in ‘D9320’ plants, which indicated that POD responds to PM in a low-residue-abundance cucumber cultivar. The enzyme activity of the ‘D0351’ *CsMAPEG*(−)-overexpressing plants was lower than that of the ‘D0351’ wild-type plants at the various time points after PM treatment. After PM treatment, the ‘D9320’ *CsMAPEG*(+)-overexpressing plants exhibited higher activity than the ‘D9320’ wild-type plants at all tested time points with the exception of 48 h. These results indicated that the alternation in *CsMAPEG* expression changed the POD enzyme activity in plants, *CsMAPEG* overexpression enhanced POD enzyme activity, and antisense *CsMAPEG* weakened POD enzyme activity.

**Fig. 9:**
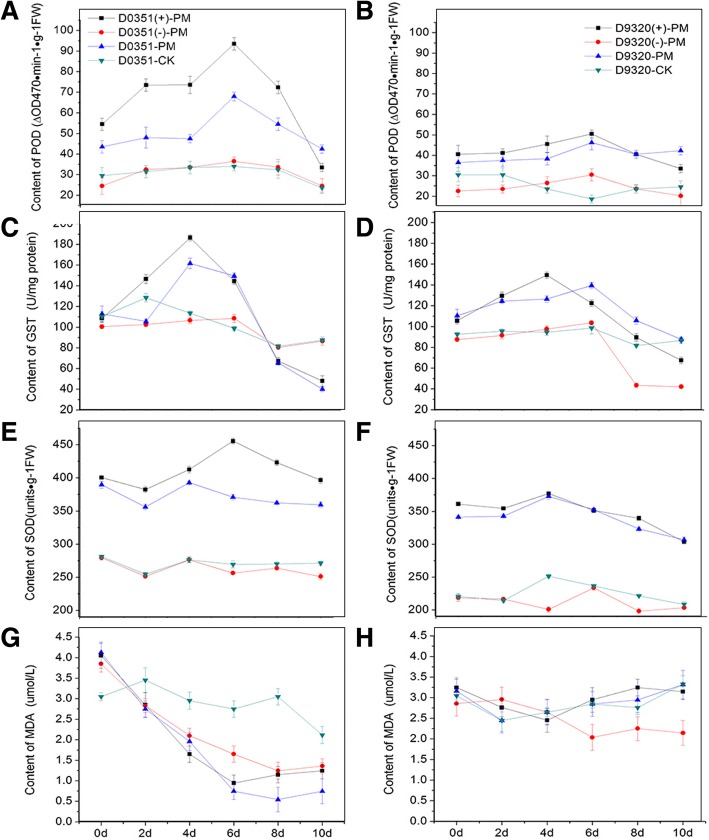
Analysis of physiological indexes under T_1_ stress of PM. **a**: ‘0351’ POD content; **b**: ‘9320’ POD content; **c**: ‘0351’ GST content; **d**: ‘9320’ GST content; **e**:‘0351’ SOD content; **f**: ‘9320’ SOD content; **g**: ‘0351’ MDA content; **h**: ‘9320’ MDA content

The change in the POD content in the T1-generation fruits was the same as that found in the T0-generation fruits (Fig. 9a and b). ‘D9320’ *CsMAPEG*(+)-overexpressing plants showed a maximum POD value of 50.48△OD470•min-1•g-1 FW at 6 days. The maximal difference in activity between the ‘D9320’ *CsMAPEG*(+)-overexpressing and wild-type plants after PM treatment appeared on day 4, and the POD activity of the *CsMAPEG*(+)-overexpressing plants was 1.19-fold higher than that of the wild-type plants. The POD activity of the ‘D0351’ *CsMAPEG*(−)-overexpressing plants was similar to that of the wild-type plants after PM treatment, although the POD content in these fruits was lower than that of the ‘D0351’ *CsMAPEG*(+)-overexpressing plants, and this difference was highly significant.

#### GST analysis of *CsMAPEG*-overexpressing plants

As shown in Fig. 8c and d, the GST content of the ‘D0351’ wild-type plants treated with distilled water first increased, reaching a maximum value of 191.14 U•mg-1 on day 6, and then decreased. However, the GST content of the ‘D9320’ wild-type plants treated with distilled water decreased continuously. The GST contents in the two cucumber genotypes showed different trends; thus, GST might be related to the detoxification of PM in cucumber fruits. After PM treatment, the GST content of ‘D0351’ *CsMAPEG*(−)-overexpressing plants was lower than that of the wild-type plants at all tested time points with the exception of day 6. In addition, after PM treatment, the GST content of the ‘D9320’ *CsMAPEG*(+) plants at each time point was significantly higher than that of the ‘D9320’ wild-type plants, which indicated that the GST content was affected by the expression of *CsMAPEG*. Plants overexpressing *CsMAPEG* exhibited a high GST content, whereas plants overexpressing antisense *CsMAPEG* showed a low GST content.

The GST content in the T1 fruits from ‘D0351’ *CsMAPEG*(−)-overexpressing plants was lower than that in ‘D0351’ wild-type plants at most time points after PM treatment. The GST content of ‘D9320’ *CsMAPEG*(+)-overexpressing plants was higher than that of the wild-type plants at most tested time points after PM treatment, and the general trend was similar to that found for the T0-generation plants (Fig. 9c and d).

#### SOD analysis of *CsMAPEG*-overexpressing plants

As shown in Fig. 8e and f, the SOD content of the ‘D0351’ wild-type plants treated with distilled water increased slightly starting from day 2 and reached a maximum of 264.98 U•g-1 FW on day 4, whereas that of the ‘D9320’ wild-type treated with distilled water showed a continuous decline. These findings indicated that SOD was directly involved in the metabolic response to the pesticide PM, and the difference in its content might be one of the reasons for the difference in PM residue abundance found between fruits of the two varieties. After PM treatment, the SOD contents of the ‘D0351’ *CsMAPEG*(−)-overexpressing plants were lower than those of the ‘D0351’ wild-type plants at all tested time points. The SOD contents of the ‘D9320’ *CsMAPEG*(+)-overexpressing plants at all tested time points after PM treatment were higher than those of the ‘D9320’ wild-type plants. These results showed that the expression of *CsMAPEG* affected the SOD content in cucumbers.

The SOD content in the T1-generation plants was lower compared with that of the T0-generation plants at different time points, although the overall trend remained unchanged. After PM treatment, the SOD content of the ‘D0351’ *CsMAPEG*(−)-overexpressing plants was lower than that of the ‘D0351’ wild-type plants at each tested time point. In addition, the SOD content of the ‘D9320’ *CsMAPEG*(+)-overexpressing plants was basically the same as that of the ‘D9320’ wild-type plants after PM treatment (Fig. 9e and f).

#### MDA analysis of *CsMAPEG*-overexpressing plants

As shown in Fig. 8g and h, the change in the MDA content after PM exposure showed differences between the ‘D0351’ and ‘D9320’ varieties. The MDA content in ‘D0351’ decreased rapidly, reached the lowest value after 6 days, and then slightly increased. In ‘D9320’ plants, the MDA content decreased slightly during the first 2 days and then increased substantially. ‘D0351’ and ‘D9320’ plants were subjected to four different treatments, namely, the transfection of sense or antisense constructs and the spray administration of distilled water or PM. The results showed that the MDA contents in the same variety were similar and that the differences in the MDA content following the different treatments were very small. The trend obtained for the T1-generation plants was the same as that found for the T0-generation plants, and the MDA content showed slight differences (Fig. 9g and h). These findings indicated that the MDA content in cucumber fruits was only affected by cultivar differences and that *CsMAPEG* had no effect on the content of MDA. Thus, although MDA is involved in the stress response, its metabolic effect was not significant after PM treatment.

## Discussion

A transcriptome analysis showed that exogenous PM can activate the GST metabolism pathway in cucumber, including the MAPEG family, which is a newly discovered microsome protein superfamily. Most members of this family participate in the metabolism of exogenous lipids and have specific detoxification and antioxidation functions. For example, mGST1, which is a phase II drug-metabolizing enzyme that belongs to the MAPEG family, has the following two functions in vivo: as a glutathione S-transferase, it catalyzes the metabolism of lipophilic and electrophilic substrates and decreases the toxicity and increases the excretion of the latter in vitro, and as a GST-dependent peroxidase, it catalyzes the combination of oxidative stress products and GSH and exerts anti-lipid peroxidation effects. Thus, this protein plays an extremely important role in the stability of the internal plant environment [28].

Analyzing the expression patterns of genes is important for the elucidation of gene functions. A significant difference in the expression patterns of *CsMAPEG* was observed between cucumbers with different genotypes. Following PM exposure, the expression of this gene was significantly upregulated at all time points in ‘D0351’ and downregulated at most time points in ‘D9320’. The expression of *CsMAPEG* in the roots, stems, leaves and fruits of ‘D0351’ was significantly higher than that in the same organs of ‘D9320’ at the same time points. The stems and leaves were sprayed with PM, and the results showed that these organs are involved in PM transport, as reflected by the finding that the most obvious differences in expression were found in these organs. These results are consistent with the findings obtained in a transcriptome sequencing analysis performed in a previous study [12]. Our findings indicated that *CsMAPEG* was specifically upregulated in the low-residue-abundance cucumber cultivar ‘D0351’ and responded positively to PM exposure. Thus, this gene is an important mechanism for the differences in the residue abundances between the two cucumber genotypes. By analyzing the expression of *CsMAPEG* in different tissues and organs of ‘D0351’ and ‘D9320’ after PM stress, we found that *CsMAPEG* was constitutively expressed and showed obvious tissue differences, particularly in the leaves, stems and fruits. The *CsMAPEG* expression levels in different tissues and organs were ordered as follows: fruits>leaves>stems>roots. Previous research analyzed the expression patterns of *CsABC19* in ‘D0351’ and ‘D9320’ under PM stress, and the results showed that the *CsABC19* gene was mainly expressed in ‘D0351’ [13], and this previous study revealed similar expression patterns for the *CsMAPEG* and *CsABC19* genes. Another study showed that the *CsABC19* gene was mainly expressed in cucumber fruits, and the results of the tissue-specific analysis performed in the experiment also demonstrated that fruits exhibited the highest relative expression. Based on the above-presented results, *CsMAPEG* can actively respond to PM stress and might promote tolerance to PM and participate in the detoxification of pesticides.

The 2000-bp promoter sequence upstream of the *CsMAPEG* starting codon was obtained from the cucumber genome database, and the promoter sequence of *CsMAPEG* was analyzed. Many light-response elements, which are regulated by light signals, were found in the promoter region of this gene. Many hormone-responsive elements, such as the TCA element, the GARE motif, and ERE cis-regulatory elements, can regulate SA, GA, ethylene and other hormones. Hormones can play an important role in the adaptation and signal transduction of the plant stress response. SA can induce an increase in membrane protective enzymes (mainly SOD and POD) in cucumber leaves, and these enzymes scavenge oxygen free radicals and enhance the antioxidant capacity of plants [29]. Under stress conditions, the GA synthesis system can be activated to synthesize high levels of GA and thereby enhance the ability of plants to resist stress [30]. JA is an endogenous growth regulator in higher plants. During stress resistance, the signal molecules inducing the expression of resistance genes are closely related to plant resistance [31]. In this study, we found that the expression patterns of *CsMAPEG* showed significant differences between ‘D0351’ and ‘D9320’ after SA and GA induction. ‘D0351’ was more sensitive to hormone induction than ‘D9320’, and this finding might be related to the low PM residue abundance in ‘D0351’. However, further experiments are needed to confirm whether the resistance of cucumber to PM stress can be enhanced by regulating the hormone pathway and subsequently the metabolism of PM. The *CsMAPEG* gene sequence also contains many defense and stress-induced response regulatory elements, which might play an important role in plant resistance to PM. Stress resistance can induce plant resistance, and new varieties with varying levels of stress and disease resistance have been screened under stress conditions. Under drought and low temperature stress conditions, the expression patterns of *CsMAPEG* in ‘D0351’ and ‘D9320’ were the same, and no difference was found among the cultivars. However, the relative expression of *CsMAPEG* in ‘D0351’ was significantly higher than that in ‘D9320’, and ‘D0351’ was more responsive to drought and low temperature stress than ‘D9320’. The expression of *CsMAPEG* after Cor stress was significantly higher than that obtained after the other two stress treatments, which suggested that this gene is responsive to *Corynespora* leaf spot and might be involved in disease resistance. It has been confirmed that ‘D9320’ shows high resistance to Cor, and the expression of *CsMAPEG* in ‘D9320’ was higher than that in ‘D0351’ under Cor stress. Thus, *CsMAPEG* might play a role in resistance to *Corynespora cassiicola*, but whether the gene is a disease-resistance gene and the specific regulatory mechanism of cucumber resistance to this disease need to be further verified.

Because fruits constitute the edible part of cucumber plants and exhibit the highest *CsMAPEG* expression, fruits from the T0- and T1-generation plants with the same growth potential and similar *CsMAPEG* expression were selected for the detection of PM residues. A comparative analysis of the data shown in Figs. 6 and 7 reveals that the PM residue abundances in the ‘D0351’ T0-generation, T1-generation and wild-type plants were lower than those in the ‘D9320’ plants, which indicated that ‘D0351’ is a cucumber variety with a lower PM residue abundance compared with ‘D9320’. This result is consistent with those reported by Liu [9] and Wu [2]. Based on the above-described analysis (Fig. 4), *CsMAPEG* expression in ‘D9320’ increased rapidly starting at 12 h. After 12 h, the PM residue abundance in *CsMAPEG*(+)-overexpressing plants was significantly or extremely significantly lower than those in the *CsMAPEG*(−)-overexpressing and wild-type plants, which indicated that the PM residue level was negatively correlated with the *CsMAPEG* gene expression level. Zhao Wen et al. studied the rate of change in the pesticide residue abundance in fruits. The highest rate of change in the pesticide residue abundance in cucumber fruits was detected at 24 h [11]. In the T0 generation, the most significant differences among *CsMAPEG*(+)-overexpressing, *CsMAPEG*(−)-overexpressing and wild-type plants were found at 24 h. After 24 h, the residue abundances of the *CsMAPEG*(+)-overexpressing plants showed a lower decrease. The T1-generation plants showed the most significant difference at 72 h, and the *CsMAPEG*(+)-overexpressing plants showed obvious upward and downward trends at each time point. These results might be due to differences in the genetic process of T1-generation plants. *CsMAPEG* can effectively reduce the PM residue abundance in ‘D0351’ and ‘D9320’ T1-generation plants, which indicated that the gene exhibits certain genetic stability. *CsMAPEG* can respond to PM stress, and *CsMAPEG*(+) overexpression plays an important role in reducing the PM residue abundance. This gene can effectively reduce the abundance of PM residues and might be related to detoxification and metabolism in cucumber. The transfection of antisense genes increased the PM residue abundance in fruits, which indicated that antisense genes play an inverse regulatory role. The transfection of the antisense gene might have inhibited degradation- and metabolism-related enzymes or genes and affected the detoxification metabolism of PM in cucumber fruits. This result is consistent with those reported by Liu [14].

In our study, the transfer of *CsMAPEG*(+) into cucumber increased the SOD and POD contents in cucumber fruits, and the transfer of antisense *CsMAPEG*(−) into cucumber resulted in significantly lower SOD and POD contents compared with those found in the corresponding wild-type control. These findings indicated that *CsMAPEG* affected the SOD and POD contents in cucumber. The SOD and POD contents could be regulated by increasing the expression of *CsMAPEG* and might induce the accumulation of H_2_O_2_ in cucumber fruits. The metabolism of PM and the effective reduction of pesticide residues in cucumber might be improved by the reduction of H_2_O_2_ to H_2_O. The results were consistent with those reported by Chaitanya [32], Mazorra [33], and Davies [34] as well as those obtained in other studies on POD activity after abiotic stress. Our results showed that the variation in the MDA content in plants of the same variety was similar and that the difference in the MDA content was very small. The MDA content in cucumber fruits was greatly affected by the variety, although *CsMAPEG* had little effect on the content of MDA. Moreover, MDA is involved in the stress response, and detoxification is the first important function of GST identified in organisms. Plants can self-detoxify toxic chemicals in the body, and GST plays an important role in this process [22]. In addition, the GST content in cucumber increased significantly under abiotic stresses, and this finding indicated that GST can also act as a cellular signal in plant stress tolerance. The research results showed that the GST trends in the high- and low-residue-abundance cultivars were different and that GST might be involved in the detoxification metabolism of PM in cucumber fruits. *CsMAPEG* overexpression affected the GST content in cucumber, and the opposite trend was found for antisense *CsMAPEG*. These findings indicated that the transfer of *CsMAPEG*(+) into cucumber could increase the GST levels and that GST could detoxify cucumber fruits and reduce the damage caused by PM. These results correspond to the upregulation of GST-related genes and the significant enrichment of the GSH metabolism pathway detected in the analysis of gene expression profiles [12]. PM stress might activate the GSH metabolism pathway in cucumber. The role of *CsMAPEG* in the pathway could enhance the activity of protective enzymes in the membrane system, enhance the antioxidant capacity of cucumber and stabilize the internal environment of cucumber. This protein can also increase the GST content, and GST catalyzes the metabolism of PM, thereby reducing the toxicity of PM, increasing its discharge in vitro, and reducing the damage caused by PM residues.

## Conclusions

In summary, the subcellular localization of *CsMAPEG* was found to be cytoplasmic, which identified this protein as a cytoplasmic protein. *CsMAPEG* displayed differences between varieties and tissues, was strongly expressed in ‘D0351’ and showed its highest expression in fruits. *CsMAPEG* can respond to SA, GA and Cor and might play an important regulatory role in the response of plants to abiotic and biological stresses. The overexpression of *CsMAPEG*(+) in cucumber effectively reduced the PM residue abundance, increased the activities of protective enzymes (POD and SOD) in the cucumber inner membrane system and increased the activity of GST, which has detoxification functions. The transfer of antisense *CsMAPEG*(−) into cucumber had the opposite effects. In brief, the results showed that *CsMAPEG* plays an important role in reducing the PM residue abundance in cucumber. This study provides a valuable foundation for further study of the molecular mechanisms responsible for the low accumulation of PM residues in cucumber. Ultimately, a greater understanding of these molecular mechanisms will enable targeted breeding strategies for the development of cucumber varieties with low pesticide residues and increased value.

## Methods

### Plant materials and stress treatments

The homozygous cucumber lines ‘D0351’ (with a low PM residue abundance) and ‘D9320’ (with a high PM residue abundance) were selected as the experimental materials [2, 9]. Seeds of these lines were provided by the cucumber research group at Northeast Agricultural University, Harbin, China. ‘D0351’ and ‘D9320’ seeds were planted in soil at the Biotron in March 2013 under the following conditions: day/night temperatures of 28/18 °C, 12-h day/12-h night cycle, and 70% relative humidity. Conventional barrel-and-frame cultivation methods were adopted for unified management. Once the cucumber was ripe at the 10th node, the plants were sprayed with 400x PM solution until the surface of the leaves and fruits began to drip, and the control group was sprayed with the same amount of distilled water. Samples were obtained 1, 6, 12, 24, 48 and 72 h after treatment, frozen in liquid nitrogen and stored at − 80 °C for gene expression analysis.

Three true-leaf seedlings were used to determine the changes in *CsMAPEG* expression after treatment with SA (foliar spraying with 0.5 mmol/L SA [35]), JA (foliar spraying with 100 μmol/L JA), GA (foliar spraying with 100 μmol/L GA [14]), drought stress (seedlings were irrigated with 50 mL of 40% PEG4000, and leaves were harvested 8 days after treatment), *Corynespora cassiicola* Wei (Cor) stress (disease stress; seedlings were sprayed with 1 × 10^5^ colony-forming units/mL Cor, and leaves were harvested 24 h after treatment) and cold (low-temperature) stress (the day/night temperatures were set to 10 ± 0.5 °C/5 ± 0.5 °C, the illumination time was 16 h, the light intensity was 4000 lx, and the stress was applied for 10 days). Five plants with identical growth were selected as replicates of each treatment, and leaves were harvested 24 h after treatment, frozen with liquid nitrogen and stored at − 80 °C for analysis of the expression patterns induced by external factors.

T0-generation *CsMAPEG*(+) and *CsMAPEG*(−) transgenic cucumber plants with similar expression levels were sprayed with 4 mM PM solution. Leaves were harvested 1, 6, 12, 24, 48 and 72 h after treatment. Wild-type ‘D9320’ plants were sprayed as controls. Seeds of the T1-generation plants were obtained through self-crossing of the T0-generation lines. The T1-transgenic plants were treated using the same protocols as those used for the T0-generation plants.

### Cloning and bioinformatics analysis of *CsMAPEG*

The full-length coding sequence (CDS) of *CsMAPEG* was obtained by BLAST alignment using the cucumber genome database (http://www.icugi.org/). Primer Premier 5.0 software (PREMIER Biosoft International, CA, USA) was used to design the primers for cloning the CDS of CsMAPEG (geneIDCsa5G409710.1): *CsMAPEG-*F, ATGGCCGCAATCCAGCTTCTC; and *CsMAPEG-*R, TTAATGACGAAGAAGCTTAACACCGAAC. PCR amplification was performed using ‘D0351’ cDNA as the template and the following temperature program: 94 °C for 5 min, 35 cycles of 94 °C for 30 s, 58 °C for 30 s and 72 °C for 30 s and 72 °C for 10 min. The amplified PCR products were detected by agarose gel electrophoresis, and a colloid recovery kit (TransGen Biotech) was used to recover the target bands. The recovered fragments were attached to the T3 vector (TransGen Biotech) using the following linkage system: 4 μL of the target gene and 1 μL of the T3 ligase were reacted at room temperature (20 °C–37 °C) for 5 min. After the reaction, the sample was transformed into *Escherichia coli* DH5α competent cells by thermal shock, and the cells were then incubated overnight on LB agar plates containing X-Gal, IPTG and Amp at 37 °C. Single white bacterial colonies were isolated and sent for sequencing at GENEWIZ.

The *CsMAPEG* protein sequence was analyzed using the Conserved Domain Database (CDD) of NCBI (https://www.ncbi.nlm.nih.gov/), and ProtParam (https://web.expasy.org/cgi-bin/protparam/protparam) was used to assess the physicochemical properties of the amino acids. The ProtScale website (https://web.expasy.org/protscale/) was used to predict the hydrophilicity of the gene-encoded proteins. Online prediction of the protein transmembrane structure was performed using TTMHMM Server v.2.0. The signal peptide prediction of cucumber proteins was performed using SignalP 4.1 Server (http://www.cbs.dtu.dk/services/SignalP/), and the protein secondary structure was predicted using the SPOMA online tool (https://npsa-prabi.ibcp.fr/cgi-bin/npsa_automat.pl? Page = npsa_sopma.html). The phylogenetic tree was constructed using the neighbor-joining method with MEGA 5.2 software.

### Real-time quantitative reverse transcription-polymerase chain reaction (qRT-PCR) analysis

Total RNA from 100 mg of fresh cucumber leaves was extracted using the TRIzol reagent (Invitrogen™) and used for qRT-PCR analysis after ground in a sterilized grinding bowl with liquid nitrogen, and the RNA purity was detected by gel electrophoresis. A reverse transcription kit (Toyobo, Japan) was used for the reverse transcription of RNA into single-stranded cDNA, and the products were stored at − 20 °C for further analysis. The following primers were designed using the online tool at https://www.genscript.com/tools/real-time-pcr-tagman-primer-design-tool: *CsMAPEG-*qF, CGCGCTCGCAAGAAGTATAA; and *CsMAPEG-*qR, GAAGCGGTAAGGCAAGGATG. qRT-PCR was performed using SYBR Green Master Mix (Toyobo, Japan), and the reaction system consisted of 10 μL of SYBR Green PCR Master Mix, 0.5 μL of the upstream primer, 0.5 μL of the downstream primer, 2 μL of cDNA template, and ddH_2_O to a total volume of 20 μL. The amplification conditions were as follows: denaturation at 95 °C for 10 min followed by 40 cycles of 95 °C for 15 s and 55 °C for 15 s. Relative quantification of gene expression was performed using CsEF1α [36] (GenBank accession number: XM_004138916) as the control (CsEF1a-qrF: CCAAGGCAAGGTACGATGAAA, CsEF1a-qrR: AGAGATGGGAACGAAGGGGAT). Four replicates of each treatment were used. A melting-curve analysis was performed after the amplification was completed. The 2-ΔΔCT method [37] was used to analyze the real-time qPCR results.

### Sequence analysis of the *CsMAPEG* promoter

The 2000-bp promoter region upstream of *CsMAPEG* was obtained from the cucumber gene database (http://www.icugi.org/), and the cis-acting elements in the promoter region were analyzed using the online tool PlantCARE (http://bioinformatics.psb.ugent.be/webtools/plantcare/html/) [38].

### *CsMAPEG* subcellular localization

The following primers were designed with XmaI and BamHI restriction sites: *CsMAPEG*-LF, CGGGATCCATGGCCGCAATCCAGCTTCTC; and *CsMAPEG*-LR, CCCCCCGGGTAATGACGAAGAAGCTTAACACCGAAC. The *CsMAPEG* open reading frame without a stop codon was amplified. The pEASY-T3-*CsMAPEG* fusion expression vector and pGII-eGFP transient expression vector were digested by rapid digestion with the endonucleases XmaI and BamHI, and the purified product was recovered by gel electrophoresis and ligated using the T4 ligase to obtain the fusion expression vector 35S-*CsMAPEG*-eGFP. This construct was transformed into *E. coli* cells and identified. The empty pGII-EGFP vector was used as a negative control. The 35S-*CsMAPEG*-eGFP and empty pGII-EGFP vector plasmids were transfected into isolated *Arabidopsis* protoplasts [39]. The subcellular localization in protoplasts was observed using a TCS SP2 confocal spectral microscope imaging system (Leica, Germany). GFP fluorescence was observed at an excitation wavelength of 488 nm and an emission wavelength of 530 nm.

### Transformation of cucumber

The full-length primers *CsMAPEG*-F and *CsMAPEG*-R were used to amplify the target gene by PCR with the pEASY-T3-*CsMAPEG* plasmid as the template. pCXSN-1250 is a plant expression vector that can be used for TA cloning [40]. The PCXSN-1250 vector was digested with XcmI. T4 ligase was used to ligate the target fragment and PCXSN-1250 vector, and the construct was then transformed into *E. coli*. The overexpression vector pCXSN-*CsMAPEG*(+) and the antisense expression vector pCXSN-*CsMAPEG*(−) were constructed, and the directionality of the target gene within the vector was confirmed by sequencing. The recombinant plasmid was transferred into *Agrobacterium tumefaciens* LBA4404 using the freeze-thaw method. The overexpression vector pCXSN-*CsMAPEG*(+), the antisense expression vector pCXSN-*CsMAPEG*(−), and empty vectors were simultaneously transferred into ‘D0351’ and ‘D9320’ by infestation of the cotyledon nodes of cucumber. Plants transformed with the empty vector were used as controls. After coculture, MS medium containing 1.0 mg/L glyphosate was used for screening and differentiation. After approximately 20 days, the cotyledonary node began to differentiate, and differentiated buds were observed. Once the differentiated buds matured, they were cut off and transferred to rooting medium. After the main and fibrous roots grew, they were transplanted and domesticated in an incubator [41]. The domesticated transgenic seedlings were planted in a greenhouse, managed uniformly and pollinated to obtain the T1 seeds. The DNA of transgenic cucumber was extracted using the CTAB method. The sequence of the pCXSN vector was used for the primers. The transgenic plants (both the T0 and T1 plants) were identified by PCR and qPCR. The PCR system consisted of 0.5 μL of upstream primer (20 μmol L-1), 0.5 μL of downstream primer (20 μmol L-1), 2 μL of 10× PCR buffer (containing 20 mmol L-1 Mg2+), 2 μL of dNTPs (10 mmol L-1), 0.2 μL of Taq enzyme (2 U), 2 μL of cDNA template, and ddH2O to a total volume of 20 μL.

### Determination of remnant PM

A mixture of the cucumber leaf sample (12.5 g) with 25 mL of acetonitrile was homogenized with a high-speed homogenizer (Heidolph Silent Crusher-M®) for 2 min at 15,000×g and incubated at room temperature for 0.5 h. The homogeneous acetonitrile was extracted into a centrifuge tube containing 3 g of NaCl, rotated on a vortex for 1 min and centrifuged for 5 min at 5000×g. Five milliliters of the supernatant was dried with a sample concentrator (Termovap) at 60 °C, and 2.5 mL of acetone was added. The mixture was then filtered through a 0.22-μm polypropylene filter. After the filtration membrane solution was clarified, the filter membrane solution was allowed to stand for several minutes to observe the presence of turbidity; if particles were present, the solution was passed through the membrane again [42] until no particles remained. An Agilent 7890B-5977A gas chromatography system (Agilent Technologies) equipped with a capillary column (HP-5MS, 30 m × 0.25 mm × 0.25 μm) was used to analyze the level of PM residues. The parameters were as follows: temperature program, maintained at 70 °C for 5 min, increased to 150 °C at a rate of 25 °C/min, increased to 200 °C at a rate of 3 °C/min, increased to 280 °C at a rate of 20 °C/min and maintained at 280 °C for 10 min; sample inlet, 250 °C; non-split injection; flow rate, 1.0 mL/min; transmission line, 250 °C; four-stage rod, 150 °C; and ion source, 230 °C [2].

### Detection of physiological and biochemical indexes

A mixture of 0.5 g of fresh cucumber leaves and 3 mL of phosphate buffer (pH 7.8) was added to an ice bath and centrifuged for 20 min at 10,500 rpm. This reaction liquid was stored at 4 °C for the determination of physiological and biochemical indexes.

POD activity was determined using the guaiacol method [43]with minor modifications. Briefly, 112 μL of guaiacol was added to 200 mL of phosphate buffer (pH 6.0), and the mixture was heated until the sufficient dissolution was detected. Subsequently, 19 μL of H_2_O_2_ (30%) was added, and the resulting reaction mixture and stored at 4 °C. Twenty microliters of the reaction mixture was combined with 3 mL of the reaction liquid to obtain the sample mixture, and 20 μL of phosphate buffer mixed with 3 mL of the reaction liquid was used as the control. The samples were placed in an ultraviolet-visible spectrophotometer (Shimadzu, Japan), and the OD value at 470 nm/min (OD values after 0, 1, 2, and 3 min) was recorded.

SOD activity was analyzed using the NBT method [44] with minor modifications. Reaction mixtures of H_2_O, phosphate buffer, Met, NBT, EDTA-Na_2_ and lactochrome were prepared at a proportion of 5:30:6:6:6:6. Subsequently, 50 μL of the reaction mixture was mixed with 3 mL of the reaction liquid. In addition, 50 μL of phosphate buffer mixed with 3 mL of the reaction liquid was used as the control group, and this control group was divided into two subgroups: control group 1 was treated in the dark, and control group 2 was treated under normal light conditions. The absorbance at OD560 was determined using an ultraviolet-visible spectrophotometer (Shimadzu, Japan).

MDA was detected as described in a previous study [2]. Briefly, 2 mL of TBA (0.67%) was added to 1 mL of the reaction liquid, and 2 mL of TBA (0.67%) mixed with 1 mL of distilled water was used as the control group. The orifice of the test tube was sealed to avoid liquid volatilization. After 15 min of incubation in a boiling water bath, the reaction was cooled and introduced into a centrifugal tube. The samples were centrifuged for 20 min at 4000 rpm and then detected at 600, 532, and 450 nm using an ultraviolet-visible spectrophotometer (Shimadzu, Japan).

GST activity was measured based on the method described by Wang Jitao [45] using a kit purchased from the Nanjing Institute of Bioengineering.

### Statistical analysis

All data measurements were replicated at least three times. The data were subjected to statistical analyses using the data processing system Origin 8.0 and DPS 9.5. The data are expressed as the means ± SDs. The significance of the differences between the treatment and control groups was confirmed by Student’s t-tests. The data were analyzed by analysis of variance (*p* < 0.05 and *p* < 0.001 indicate significant and extremely significant differences, respectively).

## Additional files

Additional file 1: **Table S1.** Locations and sequences of cis-elements in the promoter regions of the *CsMAPEG* genes. (DOC 50 kb)

Additional file 2: **Table S2.** All sequences data in Additional file 10: Figure S8. (DOC 258 kb)

Additional file 3: **Figure S1.** The conserved domain of *CsMAPEG* coding protein. (PNG 2 kb)

Additional file 4: **Figure S2.** Protein hydrophobicity prediction of CsMAPEG. (GIF 10 kb)

Additional file 5: **Figure S3.** Signal peptide of *CsMAPEG* coding protein. (PNG 9 kb)

Additional file 6: **Figure S4.** The transmembrane region of *CsMAPEG* coding protein. (PNG 5 kb)

Additional file 7: **Figure S5.** Secondary protein structure of CsMAPEG. (PNG 1 kb)

Additional file 8: **Figure S6.** Tertiary structure of protein. (PNG 9 kb)

Additional file 9: **Figure S7.** Phylogenetic tree of *CsMAPEG*. The amino sequences were subjected to phylogenetic analysis using the neighbor-joining method in MEGA5.0 software, with 1000 bootstrap replicates. (JPG 35 kb)

Additional file 10: **Figure S8.** pCXSN-CsMAPEG genetic transformation of cucumber. A:cucumber seed; B:co-culture; C:screening culture; D:plant regeneration; E:rooting culture of resistant seedlings; F:regeneration of resistant seedlings; G:seed of transgenic plants. (PNG 720 kb)

## Data Availability

The datasets used and/or analysed during the current study available from the corresponding author on reasonable request. Materials are available by contacting the corresponding author.

## Abbreviations

cDNA: complementrary DNA
CDS: Coding sequences
Cor: *Corynespora cassiicola* Wei
GA: Gibberellin acid
GFP: Green fluorescent protein
JA: Jasmonic acid
PCR: Polymerase chain reaction
PEG4000: Polyethylene glycol 4000
PM: Propyl-[3-(dimethylamino) propyl]carbamate
qRT-PCR: Real-time quantitative reverse transcription-polymerase chain reaction
RNA: Ribonuncleic acid
RT-PCR: Reverse transcription-polymerase chain reaction
SA: Salicylic acid
Tag-Seq: High-throughput tag-sequencing
