# Supplementary material for: Expression and functional analysis of the propamocarb-related gene CsMAPEG in cucumber
Source: BMC Plant Biol. 2019 Aug 22;19:371. doi: 10.1186/s12870-019-1971-z (PMC6704574; doi:10.1186/s12870-019-1971-z)
Supplement: Supplementary file 1 — Table S1. Locations and sequences of cis-elements in the promoter regions of the CsMAPEG genes. (DOC 50 kb) [file 12870_2019_1971_MOESM1_ESM.doc]

| Name | Sequence |  | Function |  |  | Position |  | +/- |
| --- | --- | --- | --- | --- | --- | --- | --- | --- |
| 3-AF1 binding site | AAGAGATATTT | Photoresponse element | | |  | 566 |  | + |
| ARE | TGGTTT | Anaerobic induced cis acting regulatory elements | | | | 695 |  | - |
| AuxRR-core | GGTCCAT | Cis acting regulatory elements participate in auxin responsiveness | | | | 1370 |  | + |
| ERE | ATTTCAAA | Ethylene responsive element | | | | 1306 |  | - |
| GARE-motif | AAACAGA | Gibberellin response element | | | | 78 |  | + |
| GT1-motif | GGTTAA | Photoresponse element | | | 419、1168、900、1473 | | | - |
| HSE | AAAAAATTTC | Cis acting elements in heat stress response | | | | 775、1239、789 |  | - |
| MBS | CGGTCA | Involvement of MYB binding sites in drought induction | | | | 1006 |  | + |
| MRE | AACCTAA | MYB binding sites are involved in photoresponse. | | | | 1340 |  | + |
| O2-site | GTTGACGTGA | Metabolism regulation of Zein | | | | 254 |  | + |
| P-box | CCTTTTG | Gibberellin response element | | | | 1389 |  | - |
| Skn-1_motif | GTCAT | Cis acting regulatory elements required for endosperm expression | | | | 268 |  | - |
| Sp1 | CC(G/A)CCC | Photoresponse element | | |  | 1135 |  | + |
| TC-rich repeats | ATTTTCTCCA | Cis acting elements involved in defense and stress response | | | | 477 |  | + |
| TCA-element | GAGAAGAATA | Cis acting elements for salicylic acid reaction | | |  | 446 |  | - |
| circadian | CAANNNNATC | Circadian rhythm control | | | 229、584、508、1490 | | | - |
