# Supplementary material for: Expression and functional analysis of the propamocarb-related gene CsMAPEG in cucumber
Source: BMC Plant Biol. 2019 Aug 22;19:371. doi: 10.1186/s12870-019-1971-z (PMC6704574; doi:10.1186/s12870-019-1971-z)
Supplement: Supplementary file 2 — Table S2. All sequences data in Additional file 10: Figure S8. (DOC 258 kb) [file 12870_2019_1971_MOESM2_ESM.doc]

| Name | | | | Sequence | | | | | | | | |
| --- | --- | --- | --- | --- | --- | --- | --- | --- | --- | --- | --- | --- |
| CsMAPEG | | | | ATGGCCGCAATCCAGCTTCTCCCCTCACAATATGGCTACGTCGTCCTCGTTCTTGTCCTCTACACCTTTCTCAATTTCTG | | | | | | | | |
|  |  |  |  | GATGGCCGGTCAGGTCGGCCGCGCTCGCAAGAAGTATAAGGTCTTTTACCCTAATTTATACGCTCTCGAATCCGACAACA | | | | | | | | |
|  |  |  |  | AGGATGCCAAACTCTTCAATTGTGTCCAGAGAGGGCACCAGAATTCGCTAGAAATGATGCCTTTGTTCTTCATGCTTATG | | | | | | | | |
|  |  |  |  | ATTTTGGGAGGAATTGGGCATCCTTGCCTTACCGCTTCCTTTGGAGTTCTCTATGTTGTGTGTCGATTCTTCTACTTCAA | | | | | | | | |
|  |  |  |  | AGGTTACGCTACCGGTGTGCCTGAAAAGCGCCTCACCATCGGGAAATTTTCTTTTCTGGCATTACTCGGATTGATGGTTT | | | | | | | | |
|  |  |  |  | GCACAATCTCGTTCGGTGTTAAGCTTCTTCGTCATTAA | | | | |  |  |  |  |
|  |  |  |  |  |  |  |  |  |  |  |  |  |
| *Cucumis melo* L.(XM 008462283.2) | | | | TGCAAAACCCTAATCCCCAATTCCAAAGATACACGATCGATCCAGTCAAGCTTCATAGTTGACCCAATCCATCTATGGCT | | | | | | | | |
|  |  |  |  | TGGAGAAGAAGTTGCCGACTGTACTCCCTTACCAATAAGATCCCCATTCCATCTCCTTCATTCCTCACCTCCTTCTCCCG | | | | | | | | |
|  |  |  |  | CAATGGCCGCAATCCAGCTTCTCCCCTCAGAATATGGCTACGTCGTCCTCGTTCTTGTCCTCTACTCCTTTCTCAATTTC | | | | | | | | |
|  |  |  |  | TGGATGGCCGGTCAGGTCGGCCGCGCTCGCAAGAGGTATAAGGTCTTTTACCCTAATTTATACGCTCTTGAATCCGAGAA | | | | | | | | |
|  |  |  |  | CAAGGATGCCAAACTCTTCAATTGTGTCCAGAGAGGGCACCAAAATTCGCTAGAAATGATGCCTTTGTTCTTCATGCTTA | | | | | | | | |
|  |  |  |  | TGATTTTGGGAGGAATTGGGCATCCTTGCCTTAGCGCTTCCTTTGGAATTCTCTATGTTGTGTCTCGATTCTTCTACTTC | | | | | | | | |
|  |  |  |  | AAAGGTTACGCTACCGGTGTGCCTGAAAAGCGCCTCACCATCGGGAAATTTTCTTTTCTGGCATTGCTGGGGTTGATGGT | | | | | | | | |
|  |  |  |  | TTGTACAATCTCGTTGGGTGTTAAGCTTCTTCGTCAGTAAGCTTATAATCCTGTTGAGCTTGGGCTGATGTTTTAGTGTT | | | | | | | | |
|  |  |  |  | GTTCTTCATCTTCCTCGTTGCCTTGGACTTCAGTCTATGACTTTTGATTGTGTTGGTTTTTTTTGTGTTATTAAGAGTTT | | | | | | | | |
|  |  |  |  | GAATAACGGTTTTTTTTTCCCTTCCTAATCTTTGTTATAGAAGTTGTAACTTTTTACTTTAAAAAAAAAAAGAAAAAGTT | | | | | | | | |
|  |  |  |  | ATAGTAAATTTGAGACTTCTTTTATCAAATGAAGTGTAAAACGTAGATTTGTCTCTTAATTTAAGAGAGGCCTTTGAACT | | | | | | | | |
|  |  |  |  | TTCGTCATGTTCAATCCTATTTTCTTTGTAGTTATATATCTCTTTGCTCATTTGGAAGGTAGTGAATTGAGCTATACAAA | | | | | | | | |
|  |  |  |  | TGTTTTATCTGGA | |  |  |  |  |  |  |  |
|  |  |  |  |  |  |  |  |  |  |  |  |  |
| *Momordica charantia* L.（XM 022285675.1） | | | | TCACCCACGGAAATTTCTAGAATCTCGAACCCTAATCATCGATCCATTCACATTTCACAGTTGACCGATCCGATTAAAGA | | | | | | | | |
|  |  |  |  | AAACCCAATGGCTTGGAGAAAAAGTTGCTGAATAACGATAAGAACCCCAACCTTGGAGCTCTCTTGCCCTCACTCCTCAC | | | | | | | | |
|  |  |  |  | CTCCAAAAATGGCCGCAATCGAGCTTTTGCCCGCCGAATATGGCTACGTCGTCCTCGTTCTTGTTCTCTACACCTTTCTT | | | | | | | | |
|  |  |  |  | AATTTCTGGATGGCCGGCCAAGTCGGCAGAGCTCGCAAGAAGTATAAGGTGTTTTACCCGAATCTGTATGCCCTGGAATC | | | | | | | | |
|  |  |  |  | CGAAAACAAGGATGCTAAGCTCTTCAATTGCGTTCAGAGAGGACACCAGAATTCGCTTGAAATGATGCCTCTGTTCTTCA | | | | | | | | |
|  |  |  |  | TGCTCATGATTTTGGGAGGAATTAGGCATCCTTGCCTTAGCGCTTCGTTCGGTGTTCTCTACGTCGTAAGTCGGTTCTTC | | | | | | | | |
|  |  |  |  | TATTTCAGAGGTTACGCCACCGGCGTGCCTGAAAACCGCCTCACAATCGGGAAATTTTCCTTTCTGGCGGTGCTTGGTTT | | | | | | | | |
|  |  |  |  | GATGATTTGCACAATCTCTTTCGGTGTTACGCTTCTTCGCGCTTGAGCTCTGATCCTGTAGTGTTGTTTCTTCATCTTGC | | | | | | | | |
|  |  |  |  | AGCTCGTTCTCTTGGATTTTGATTTTGATTTTGATTTTGATTTAGGGTTGAAGTTGATGTAGTGTTGAATAAAGGAGATT | | | | | | | | |
|  |  |  |  | ACTGGTTTCTGATGTGGTATTGGGTTTTTATGATTGCCATATTAATTTTGCACTTTCCTTTTTA | | | | | | | |  |
|  |  |  |  |  |  |  |  |  |  |  |  |  |
| *Cucurbita moschata* （XM 023137096.1） | | | | GGAGAATTCTAGATTCTGAAACCCTAATCCCCAAATTCCACTCAGAGACGATCCGGTCATGTTTTACAGTTGACTCAACC | | | | | | | | |
|  |  |  |  | AATCAATAGTAAACTCAATGGCTTGGAGAAGAAGTTGCCGACTACACTCCCTTACCCATAAGAACCTCCTTCCATCTCCT | | | | | | | | |
|  |  |  |  | TCAATTCTCACCTCCATCTCCAAAAATGGCCGCTATCGACCTTTTCCCCAGAGAATATGGCTATGTCGTCCTTGTTCTTG | | | | | | | | |
|  |  |  |  | TCCTCTACTCCTTTTTCAATTTCTGGATGGCCGGTCAGGTCGGCCGCGCTCGGAAGAAGTATAAGGTCTTTTACCCCAAT | | | | | | | | |
|  |  |  |  | CTCTACGCTCTGGAATCTGAGAACAAGGATGCGAAGATCTTCAACTGTATCCAGAGAGGGCACCAGAATTCGCTGGAAAT | | | | | | | | |
|  |  |  |  | GATGCCTCTGTTCTTCATGCTTATGATTTTTGGAGGAATTAGGCATCCTTGTATTAGCGCTTCGTTGGGGGTTCTCTATG | | | | | | | | |
|  |  |  |  | TCGTGGCTCGGTTCTTCTACTTCAGAGGTTACGCCACTGGCGTGCCTGAAAAGCGTCTCACCATCGGGAAATATTTTTTC | | | | | | | | |
|  |  |  |  | TTGGCGTTGCTTGGTCTGATGATTTGCACAATCTCGTTTGCCGTGACGCTTCTTCGTGCTTAAGGTCTGGTCCTCTAGCC | | | | | | | | |
|  |  |  |  | TTGCGTGATGTTGTAGTGTTCTTCATTTTCATCTTCATCTTCATCCTCCTCGCCCTCCTGGACTTGGATCTGCTACTTCT | | | | | | | | |
|  |  |  |  | GATTTTCAGTTTGATGAAATTGAATAAGGAAACTGACTGGTTTGTTTTCAAGTGTTTTTTCTTAATCATCTTATATTATG | | | | | | | | |
|  |  |  |  | TGGGATTAGATTTAGAACCATAGAATTATTATGCCAATTGATGAGTTAAAACATCTCTCCCGTTGACATTGTCCTGATTA | | | | | | | | |
|  |  |  |  | AACCTCCAATCAAATTAATAACACTAAGAATTATATTTTCACCACTTGAAATCTAAACATTATTATTAAAAGTAA | | | | | | | | |
|  |  |  |  |  |  |  |  |  |  |  |  |  |
| *Cucurbita moschata* （XM 023695585.1) | | | | GACGGAGAATTCTAGATTCTGAAACCCTAATCCCCAAATTCCACTCAGAGACGATCCGGTCAGGTTTTACAGTTGACTCA | | | | | | | | |
|  |  |  |  | ACCAATCAATAGTAAACTCAATGGCTTGGAGAAGAAGTTGCCGACTACACTCCCTTACCCATAAGAACCTCCTTCCATCT | | | | | | | | |
|  |  |  |  | TCTTCAATTCTCACCTCCATCTCCAAAAATGGCCGCTATCGACCTTCTCCCCAGAGAATATGGCTATGTCGTCCTTGTTC | | | | | | | | |
|  |  |  |  | TTGTCCTCTACTCCTTTTTCAATTTCTGGATGGCCGGTCAGGTCGGCCGCGCTCGGAAGAAGTATAAGGTCTTTTACCCC | | | | | | | | |
|  |  |  |  | AATCTCTACGCTCTGGAATCCGAGAACAAGGATGCTAAGATCTTCAACTGTATCCAGAGAGGGCACCAGAATTCGCTGGA | | | | | | | | |
|  |  |  |  | AATGATGCCTCTGTTCTTCATGCTTATGATTTTGGGAGGAATTAGGCATCCTTGCATTAGCGCTTCGTTGGGGGTTCTCT | | | | | | | | |
|  |  |  |  | ATGTCGTGGCTCGGTTCTTCTACTTCAGAGGTTACGCCACTGGCGTGCCTGAAAAGCGTCTCACCATCGGGAAATATTTT | | | | | | | | |
|  |  |  |  | TTCTTGGCGTTGCTTGGTCTGATGATTTGCACAATCTCGTTTGCTGTGACGCTTCTTCGTGCTTAAGGTCTGGTCTTCTA | | | | | | | | |
|  |  |  |  | GCTTTGCTTTGCGTGATGTTGTAGTGTTCTTCATCTTCCTCGCCCTCTTGGCCTTGGATCTGCTACTTCTGATTTTGGGT | | | | | | | | |
|  |  |  |  | TTGATGAAATTGAATAATGAAATTGACTGGTTTGTGATCAAGTGTTTTTTTCTTAATCATCTTCTTATATTATGTGGGAT | | | | | | | | |
|  |  |  |  | TAGATTTAGAACCATAGAATCATTATACCAATTGATGAGTTAAAA | | | | | |  |  |  |
|  |  |  |  |  |  |  |  |  |  |  |  |  |
| *Cucurbita moschata* （XM 023083281.1） | | | | GGAGAATTCTAGATTCTGAAACCCTAATCCCCAAATTCCACTCAGAGACGATCCGGTCACGTTTGACAGTTGACTCAACC | | | | | | | | |
|  |  |  |  | AATCAATAGTAAACTCAATGGCTTGGAGAAGAAGTTGCCGACTACACTCCCGTCCCCATAAGAACCTCCTTCCATCTCCT | | | | | | | | |
|  |  |  |  | TCAATTCTCACCTCCATCTCCAAAAATGGCCGCTATCGACCTTCTCCCCAGAGAATATGGCTATGTCGTTCTTGTTCTTG | | | | | | | | |
|  |  |  |  | TCCTCTACTCCTTTTTCAATTTCTGGATGGCCGGTCAGGTCGGCCGCGCTCGCAAGAAGTATAAGGTCTTTTACCCCAAT | | | | | | | | |
|  |  |  |  | CTCTACGCTCTGGAATCTGAGAACAAGGATGCTAAGATCTTCAACTGTATCCAGAGAGGGCACCAGAATTCGCTGGAAAT | | | | | | | | |
|  |  |  |  | GATGCCTCTGTTCTTCATGCTTATGATTTTGGGAGGAATTAGGCATCCTTGCATTAGCGCTTCGTTGGGGGTTCTCTACG | | | | | | | | |
|  |  |  |  | TCGTGGCTCGGTTCTTCTACTTCAGAGGTTACGCCACTGGCGTGCCTGAAAAGCGTCTCACCATCGGGAAATATTTTTTC | | | | | | | | |
|  |  |  |  | TTGGCGTTGCTTGGTCTGATGATTTGCACAATCTCGTTTGCTGTGACGCTTCTTCGTGCTTAAGGTCTGGTCTTCTAGCT | | | | | | | | |
|  |  |  |  | TTGCTTTGCGTGATGTTGTAGTGTCCTTCATCATCTTCCTCGCCCTCTTGGACTTGGATCTGCTACTTCTGATTTTGGGT | | | | | | | | |
|  |  |  |  | TTGATGAAATTGAATAATGAAATTGACTGGTTTGTGATCAAGTGTTTTTTTCTTAATCATCTTGTTATATTATGTGGGAT | | | | | | | | |
|  |  |  |  | TAGATTTAGAGCCATAGAATTATTATACCAATTGATGAGTTAAAACATCTCTCC | | | | | | |  |  |
|  |  |  |  |  |  |  |  |  |  |  |  |  |
| *Chenopodiaceae* （XM 021886329.1） | | | | GCATTGAAAAAAGACCAAGTTTATAGGAAAGTTCTAGACCCTTGTAAGAAGACCCGCTCTGTCTGAACATATCTGTTGAT | | | | | | | | |
|  |  |  |  | CAGTTTTTACAAATAAGGCAGAAAATCAGAAATTGAAGCCACATTTACAATCTCAGACACAGCTAATCCTTACGTCATCC | | | | | | | | |
|  |  |  |  | CTTACATCACCATCGAATTTTCAATCGGTCATTCAATTCATTGTGCATATAAAATACAACTAAAAACTCTGAAATCTTCG | | | | | | | | |
|  |  |  |  | ACAAACCCTAAAAAAACAAAACTTGAAGAAAAATAGTCAAATTGTAAAATTTGTTGGTCAAATGAGTGAGAAAATGGGTG | | | | | | | | |
|  |  |  |  | CAACAGGAGAAGGATTTCTTGCTAAAGAATATGGATATGTTGTTTTAGTTTTGGTTTTCTATTGCTTCTTCAATTTTTGG | | | | | | | | |
|  |  |  |  | ATGGCTTTTCAAGTCGGCAAAGCAAGAAAAAAGTATAAGGTTTATTATCCAACTCTATATGCATTGGAATCTGAGAACAA | | | | | | | | |
|  |  |  |  | GGAAGCCAAACTCTTCAATTGTGTCCAGAGAGGGCACCAGAATTCATTGGAAATGATGCCAATGTTTTTCATATTGATGA | | | | | | | | |
|  |  |  |  | TTTTGGGTGGAATGGGGCATCCTTGTATTTGTGCTGGGCTTGGTGTTGCTTACATTATTTCTCGCTATTTCTACTTCACT | | | | | | | | |
|  |  |  |  | GGCTACTCATCTGGTGATCCTGAGAAACGTCTAACTGTTGGGAAATATGGTTTCTTTGCCATGTTGGGTCTGATGATCTG | | | | | | | | |
|  |  |  |  | CTCAATTTCTTTCGGAGTCAAGCTTCTTATTGCTTGAGAAGTTGGTCTTTCCTGCAAGCTTCGCACACCAATGCTTTTTC | | | | | | | | |
|  |  |  |  | CTTACATCTTCATGTTTTATGTGTACTACTTTTGAGTAAACATTAGTTAACTTTACAAGTTTTACAGACTAAGGCTTCTA | | | | | | | | |
|  |  |  |  | ATAAATCCCATGTAATTGAGCAGTTGTTTAAAGTGAAAAAAATATACGAGGGAACTAATTTAATCTCTTTGCTGTCAAAC | | | | | | | | |
|  |  |  |  | TATGCGAATTCCTTTTACTA | | |  |  |  |  |  |  |
|  |  |  |  |  |  |  |  |  |  |  |  |  |
| *Camelina sativa* L.(XM 010513204.2) | | | | CAAACAAAATCTTTCCACGTTTTTTCTTTGGTCAACAACGATGGCCGACTGACTCAAATCATCATTTTATATTTGTTAAA | | | | | | | | |
|  |  |  |  | ACCGATGATTTAAGCCCAGTACTAGTAATCACTACTAATCACCTCAACCTCCCCAAAAAAAAAAAAGAAAGAACACACCA | | | | | | | | |
|  |  |  |  | TCCATGGCGATTACAGATTTTTTACCAAAAGAGTACGGTTATGTCGTCATCATCCTCGTCCTCTACTGTTTCCTCAACCT | | | | | | | | |
|  |  |  |  | TTGGATGGGTGGTCAAGTCGGCAAAGCTCGTAAAAGGTACAATGTGCCGTATCCAACTCTCTATGCTATAGAGTCAGAAA | | | | | | | | |
|  |  |  |  | ACAAAGATGCTAAGCTCTTTAACTGTGTTCAGAGAGGACACCAAAACTCTTTGGAGATGATGCCAATGTATTTCATACTG | | | | | | | | |
|  |  |  |  | ATGATTCTTGGTGGTATGAAGCACCCTAGTATCTGTGCTATATTAGGTTTGGTCTACAACGTTAGCCGATTCTTCTACTT | | | | | | | | |
|  |  |  |  | CAAAGGTTACGCTACTGGTGATCCCATGAAGCGTCTCACTATCGGGAAATACGGTTTCTTGGGTTTGCTAGGTCTCATGA | | | | | | | | |
|  |  |  |  | TCTGTACCATCTCGTTTGGTGTCACTCTGATCCGTTCTTGAGCCACCCTTTTGTAACGTTGATGATAGTTTCTCTGGGTT | | | | | | | | |
|  |  |  |  | GTTTTAGTCTACATTTGGAACCGGTCTTTGTAAGTTGTCCACAAAATTTGTGTGATAAGAATAATCTTTGGAGTTTTCTC | | | | | | | | |
|  |  |  |  | ATTTTGTTACTTTTACAAAAACTTTTCAAAGTTGTAATAATAAATCAATGTAGAACAATCTAATTTTCACATCCCTACGG | | | | | | | | |
|  |  |  |  | AAATATATGTTGATGAGTTTGGATCA | | |  |  |  |  |  |  |
|  |  |  |  |  |  |  |  |  |  |  |  |  |
| *Camelina sativa* L.(XM 019237942.1) | | | | ATCACTAATCACTAATCAGAAGCTCAACCTCAAAACAAAAAAAAACCATCCATGGCGATTACAGATTTTTTACCAAAAGA | | | | | | | | |
|  |  |  |  | GTACGGGTATGTCGTCATCATCCTAGTCTTCTACTGTTTCCTCAACCTCTGGATGGGTGCTCAAGTCGGCAAAGCTCGTA | | | | | | | | |
|  |  |  |  | AAAGGTACAATGTGCAGTATCCAACTCTCTATGCTATAGAGTCAGAAAACAAAGATGCTAAGCTCTTTAACTGTGTTCAG | | | | | | | | |
|  |  |  |  | AGAGGACACCAAAACTCTTTGGAGATGATGCCAATGTATTTCATACTGATGATTCTTGGTGGTATGAAGCACCCTAGTAT | | | | | | | | |
|  |  |  |  | CTGTGCTATATTGGGTTTGGTCTACAACGTTAGCCGATTCTTCTACTTCAAAGGTTACGCTACTGGTGATCCCATGAAGC | | | | | | | | |
|  |  |  |  | GTCTCACTATTGGGAAATACGGTTTCTTGGGTTTGCTAGGTCTCATGATCTGTACCATCTCGTTTGGTGTCACTCTGATC | | | | | | | | |
|  |  |  |  | CGTTCTTGAGCCACACCTTTTGTAAGGTTGATGATAGATTCTCTGAGTTAGTCTACATTGGAACCGGTCTTTGTAAGTTG | | | | | | | | |
|  |  |  |  | TCCACAAAATTTGTGTGATAAAGAATAATCTTTGGAGTTTTGTTACTTCGTTTCTTTTACATTAACTTGTCAAAGTTGTA | | | | | | | | |
|  |  |  |  | ATAATAAAAGTCAATGTAGAACAATTTAATATTCACATCCCTACGGAAATATATGTTGATCAAGAGTTTGGATCA | | | | | | | | |
|  |  |  |  |  |  |  |  |  |  |  |  |  |
| *Malus pumila* (XM 008383140.2) | | | | AATACCATCCAATCCGTTGGACCATATCCATAGTTTTACACCCCCACCTGTCGGTCAGCTCAAAAGTCGATCGATCCATA | | | | | | | | |
|  |  |  |  | AAACCATTTCCCTAATTTTCTTCCTCCCCATCGTAATCTTTCAGTTCATATCAGAAATAAAAGAAAACAAAAATGTCGTC | | | | | | | | |
|  |  |  |  | TGCAATTGAATTGCTGCCCAAAGAATACGGCTATGTAGTTCTCGCCGTCGTTCTCTACACCTTTCTCAATTTCTGGATGG | | | | | | | | |
|  |  |  |  | CTGCCCAGGTTGGACGGGCCAGAAAACGGTACAAGGTKCCATATCCGACCATGTATGCGTCGGAATCGGAGAACAAAGAT | | | | | | | | |
|  |  |  |  | GCGAAAGTGTTTAACTGTGTTCAGAGAGGGCATCAGAACTCTCTCGAAATGATGCCTATGTTCTTYGTGCTTCTGGTTTT | | | | | | | | |
|  |  |  |  | GGGAGGTCTTAAGCATCCTTGCATCTCCGCCGCCCTTGGCTTCTTCTTCGCTTTTACTCGCTACTTCTACTTCAAAGGCT | | | | | | | | |
|  |  |  |  | ACGCCACCGGTGACCCCATGAACCGCCTCGCCATCGGGAAATACGGGTTTTTGGCTATATTCGGGCTTATAGGGTGCACA | | | | | | | | |
|  |  |  |  | ATTTCGTTCGGGGTYAGTCTTCTTCTTCGAGGATAAAGAAAAGCCGAACAAAGCCCTCATCTCATACTCTCATGGCCGTG | | | | | | | | |
|  |  |  |  | GAGGGCTTGCTCGCTCTTGCTATCTCATRTGCTCTGCTAAAAGGTCCTAGGGTTTTCCTTTTGTTTGTAGTATTGGCTTT | | | | | | | | |
|  |  |  |  | TGAATAAGAAAGAGCAAGAGATAGGAAGGAAATAGTGTGTGTTGCGTTTTGTAGGTCTACTTTTGATGTCTTTATGTAAT | | | | | | | | |
|  |  |  |  | TGTAAACGTTTATGTATGAATTACATGAGTTTTATAAACGTTAAGTTAATTTTGCGTCA | | | | | | |  |  |
|  |  |  |  |  |  |  |  |  |  |  |  |  |
| *Malus pumila* (XM 008372918.2) | | | | ACCATCCAATCCATTGGACCATATCCATAGTTTTACACCCCCACCTGTCGGTCAGCTCAAAAGTCGATCGATCCATAAAA | | | | | | | | |
|  |  |  |  | CCATTTCCCTAATTTTCTTCCTCCCCATCGTAATCTTTCAGTTCATATCAGAAAAAAAAGAAAACAAAAATGTCGTCTGC | | | | | | | | |
|  |  |  |  | AGTTGAATTGCTGCCCAAAGAATACGGCTATGTAGTTCTCGYCGTCGTTCTCTACACCTTTCTCAATTTCTGGATGGCTG | | | | | | | | |
|  |  |  |  | CCCARGTTGGMCGGGCCAGAAAACGGTACAAGGTKCCATATCCGACCATGTATGCGTCGGAATCGGAGAACAAAGATGCG | | | | | | | | |
|  |  |  |  | AAAGTGTTTAACTGTGTTCAGAGAGGGCATCAGAACTCTCTCGAAATGATGCCTATGTTCTTTGTGCTTCTGGTTTTGGG | | | | | | | | |
|  |  |  |  | AGGTCTTAAGCATCCTTGCATCTCCGCCGCCCTTGGCTTCTTCTTCGCTTTTACTCGCTACTTCTACTTCAAAGGCTACG | | | | | | | | |
|  |  |  |  | CCACCGGTGACCCCATGAAMCGCCTCGCCATCGGGAAATACGGGTTTTTGGCTATATTCGGGCTTATAGGGTGCACAATT | | | | | | | | |
|  |  |  |  | TCGTTCGGGGTCAGTCTTCTTCTTCGAGGATAAAGAAAAGCCGAACAAAGCCCTCATCTCATACTCTCATGGCCGTGGAG | | | | | | | | |
|  |  |  |  | GGCTTGCTCGCTCTTGCTATCTCATGTGCTCTGCTAAAAGGTCCTAGGGTTTTCCTTTTGTTT | | | | | | | |  |
|  |  |  |  |  |  |  |  |  |  |  |  |  |
| *Pyrus spp* (XM 009371791.2) | | | | CATCCAATCCATTGGACCATATCCATAGTTTCACACCCCACCAGTCGGTCAGCTCAAAAGTCGATCAATCCATAAAACCA | | | | | | | | |
|  |  |  |  | TTTCCCTAATTTTCTTCCTCCCCATCGTAATCTTTCAGTTCATATCAGAAATAAAAGAAAACAAAAATGTCGTCTGCAGT | | | | | | | | |
|  |  |  |  | TGAATTGCTGCCCAAAGAATACGGCTATGTAGTTCTCGCCGTCGTTCTCTACACCTTTCTCAATTTCTGGATGGCTGCCC | | | | | | | | |
|  |  |  |  | AAGTTGGCCGGGCCAGAAAACGGTACAAGGTGCCATATCCGACCATGTATGCGTCGGAATCGGAGAACAAAGATGCGAAC | | | | | | | | |
|  |  |  |  | GTGTTTAACTGTGTTCAGAGAGGGCATCAGAACTCTCTCGAAATGATGCCTATGTTCCTTGTGCTTCTGGTTTTGGGAGG | | | | | | | | |
|  |  |  |  | TCTTAAGCATCCTTGCATCTCCGCCGCCCTTGGCTTCTTCTTTGCTTTTACTCGCTACTTCTACTTCAAAGGCTACGCCA | | | | | | | | |
|  |  |  |  | CCGGTGACCCCATGAACCGCCTCGCCATCGGGAAATACGGGTTTTTGGCTATATTCGGGCTTATAGGGTGCACAATTTCG | | | | | | | | |
|  |  |  |  | TTTGGGGTCAGTCTTCTTCTTCGAGGATAAAGAAAAGCCGAACAAAGCCCTCATCTCATACTCTCATGGCCGTGGAGGGC | | | | | | | | |
|  |  |  |  | TTGCTCGCTCTTGCTATCTCATGTGCTCTGCTAGAAGGTCCTAGGGTTTTCTTTTTGTTTGTAGTATTGGCTTTTGAATA | | | | | | | | |
|  |  |  |  | AGAAAGTGCAAGAGATAGGAAGGAATAAGTGTGTGTTGCGTTTTGTAGGTCTACTTTTGACGTCGATATGTAATTGTAAA | | | | | | | | |
|  |  |  |  | CTTTTATGTATGAATTACACTAGTTTATAAACATTAGGTTAATTTTGCTTCAA | | | | | |  |  |  |
|  |  |  |  |  |  |  |  |  |  |  |  |  |
| *Cerasus avium* (L.) Moench. （XM 021975259.1） | | | | GTGGGCGCCCATTACCACCAAAATAAACCTAAGTGAAGGGAACAAGAGGCTTATCTATATCCGCACAAAAAACAGAGTCG | | | | | | | | |
|  |  |  |  | GTCAGTCAAGAACAAGAAGTCAATTCATAAAACCCAATTTCCCTGATTTCACGTTCATACCCAAAAACCAAATGGCCACC | | | | | | | | |
|  |  |  |  | GCAGTTGAATTGCTCCCCAGAGAGTACGGCTATGTAGTCCTTGTTCTTGTTCTCTACACCTTTCTCAATTTCTGGATGGC | | | | | | | | |
|  |  |  |  | TGGCAAAGTAGGCAAGGCCAGAAAAATGTACAAGGTGCCTTATCCGACCCTGTACGCTTCAGAATCTGACAACAAAGATG | | | | | | | | |
|  |  |  |  | CAAAAGTTTTCAATTGTGTTCAGAGAGGCCACCAGAACTCTCTGGAATTGATGCCGTTGTTCTTTGTGCTTCTGTTATTG | | | | | | | | |
|  |  |  |  | GGAGGTCTTAAACATCCTTGCATCTCAGCTGGCCTTGGTCTGCTCTATACTGTCACCCGCTATTTCTATTTCAAAGGCTA | | | | | | | | |
|  |  |  |  | CTCCACCGGCGACCCCCAGAACCGCCTTTCCGTCGGGAAGTACGGGTTCTTGGCTCTGTTGGGGCATATTGTGTGCACAA | | | | | | | | |
|  |  |  |  | TTTCCTTTGGGGTCACTCTTCTTCTCCGAGGATAAACAGAAGTACCAAAACCCTCATGGGCGTTTGGGGCTTGCCAGTGC | | | | | | | | |
|  |  |  |  | CATCTCATATGCTGCTGCTAACTTTTGGTCCCATAGTTTGTTTTTTATTTTGTAGTGTTTGGCTTTTGAATAAGAAAGTG | | | | | | | | |
|  |  |  |  | CAAGACATAGGAAGGAGGGTGTTTCCGTTTGTAGGTTTTCATGTCTTTATGTTTATGTCTGAATGAATTGCATAATTACT | | | | | | | | |
|  |  |  |  | ATCTCATTTTATTTTCTTGTACTCGTTA | | | |  |  |  |  |  |
|  |  |  |  |  |  |  |  |  |  |  |  |  |
| *Armeniaca mume* Sieb. （XM 008246089.2） | | | | AACCCAAGTGAAAAGAACAAGAGGCTCATCTATATCCACACACACAACAGACACAGTCGGTCAGTCAAGAAGTCAATTCG | | | | | | | | |
|  |  |  |  | TAAACCCCATCTCCCTGAGTTCACGTTCATACCCAAAAACCAAATGGCCACCGCAGTTGAATTGCTCCCCAGAGAGTACG | | | | | | | | |
|  |  |  |  | GCTATGTAGTCCTTGTTCTTGTTCTCTACACCTTTCTCAATTTCTGGATGGCTGGCAAAGTAGGCAAGGCCAGAAAGAAG | | | | | | | | |
|  |  |  |  | TACAAGGTGCCTTATCCAACCCTTTACGCTTCAGAATCTGACAACAAAGATGCAAAAGTTTTCAATTGTGTTCAGAGAGG | | | | | | | | |
|  |  |  |  | CCACCAGAACTCTCTGGAATTGATGCCTTTGTTCCTTGTGCTTCTGTTATTGGGAGGTCTTAAACATCCTTGCATCTCAG | | | | | | | | |
|  |  |  |  | CTGGCCTTGGTCTGTTTTATACTGTCACCCGCTATTTCTATTTCAAAGGCTACTCCACCGGCGACCCCGAGAACCGCCTT | | | | | | | | |
|  |  |  |  | GCCGTCGGGAAGTACGGGTTCTTGGCTCTGTTGGGGCATATTGTGTGCACAATTTCCTTTGGGGTCACTCTTCTTCTCCG | | | | | | | | |
|  |  |  |  | AGGATAAAACAGAAGTAACCAAAACCCTCATGGCCGTTTGGGGCTTGCCAGTGCCATCTCATATGCTGCTGCTACTTTTT | | | | | | | | |
|  |  |  |  | TGATCCCATAGTTTCTTTTTTATTTTGTAGTGTTTGGCATTTGAATAAGAAAGTGCAAGAAATAGGAAGGAGGGTGTTTC | | | | | | | | |
|  |  |  |  | CGTTTGTAGGTTTTCATGTCTTTATGTTTTTGTGTGAATGAATTGCATAATTACTATCTCATTTTATTTTCTTGTGCGCA | | | | | | | | |
|  |  |  |  |  |  |  |  |  |  |  |  |  |
| *Armeniaca mume* Sieb. （XM 008231130.2） | | | | AAAAGAACAAGAGGCTCATCTATATCCACACACACAACAGACACAGTCGGTCAGTCAAGAAGTCAATTCGTAAAACCCAT | | | | | | | | |
|  |  |  |  | CTCCCTGAGTTCACGTTCATACCCAAAAACCAAATGGCCACCGCAGTTGAATTGCTCCCCAGAGAGTACGGCTATGTAGT | | | | | | | | |
|  |  |  |  | ACTTGTTCTTGTTCTCTACACCTTTCTCAATTTCTGGATGGCTGGCAAAGTAGGCAAGGCCAGAAAGAAGTACAAGGTGC | | | | | | | | |
|  |  |  |  | CTTATCCAACCCTGTACGCTTCAGAATCTGACAACAAAGATGCAAAAGTTTTCAATTGTGTTCAGAGAGGCCACCAGAAC | | | | | | | | |
|  |  |  |  | TCTCTGGAATTGATGCCTTTGTTCCTTGTGCTTCTGTTATTGGGAGGTCTTAAACATCCTTGCATCTCAGCTGGCCTTGG | | | | | | | | |
|  |  |  |  | TCTGTTTTATACTGTCACCCGCTATTTCTATTTCAAAGGCTACTCCACCGGCGACCCCGAGAACCGCCTTGCCGTCGGGA | | | | | | | | |
|  |  |  |  | AGTACGGGTTCTTGGCTCTGTTGGGACATATTGTGTGCACAATTTCCTTTGGGGTCACTCTTCTTCTCCGAGGATAAAAC | | | | | | | | |
|  |  |  |  | AGAAGTAACCAAAACCCTCATGGCCGTTTGGGGCTTGCCAGTGCCATCTCATATGCTGCTGCTACTTTTTTGATCCCATA | | | | | | | | |
|  |  |  |  | GTTTCTTTTTTATTTTGTAGTGTGTGGCTTTTGAATAAGAAAGTGCAAGAAATAGGAAGGAGGGTGTTTCCGTTTGTAGG | | | | | | | | |
|  |  |  |  | TTTTCATGTCTTTATGTTTTTGTGTGAATGAATTGCATAATTACTATCTCATTTTATTTTCTTGTGCGCA | | | | | | | |  |
