# Supplementary figures and images for: Expression and functional analysis of the propamocarb-related gene CsMAPEG in cucumber
Source: BMC Plant Biol. 2019 Aug 22;19:371. doi: 10.1186/s12870-019-1971-z (PMC6704574; doi:10.1186/s12870-019-1971-z)

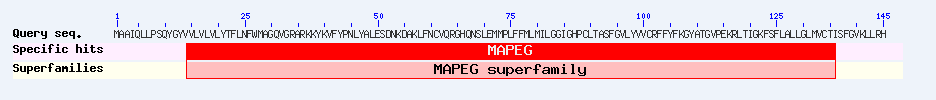

Supplement: Supplementary file 3 — Figure S1. The conserved domain of CsMAPEG coding protein. (PNG 2 kb) [file 12870_2019_1971_MOESM3_ESM.png]

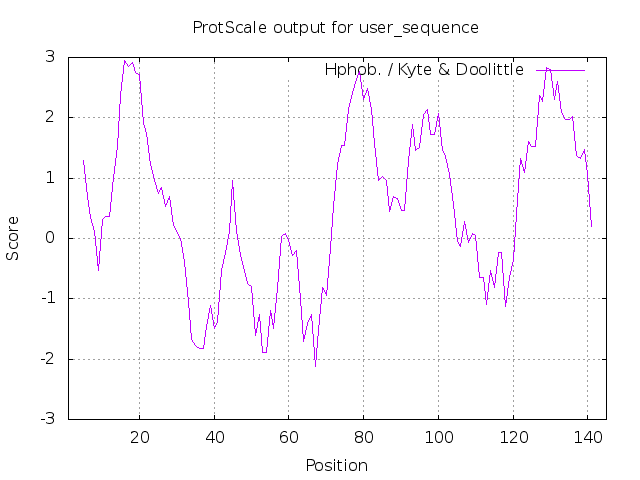

Supplement: Supplementary file 4 — Figure S2. Protein hydrophobicity prediction of CsMAPEG. (GIF 10 kb) [file 12870_2019_1971_MOESM4_ESM.gif]

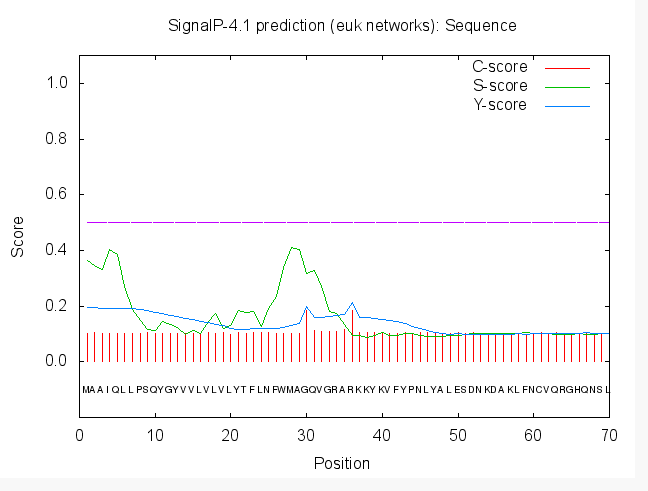

Supplement: Supplementary file 5 — Figure S3. Signal peptide of CsMAPEG coding protein. (PNG 9 kb) [file 12870_2019_1971_MOESM5_ESM.png]

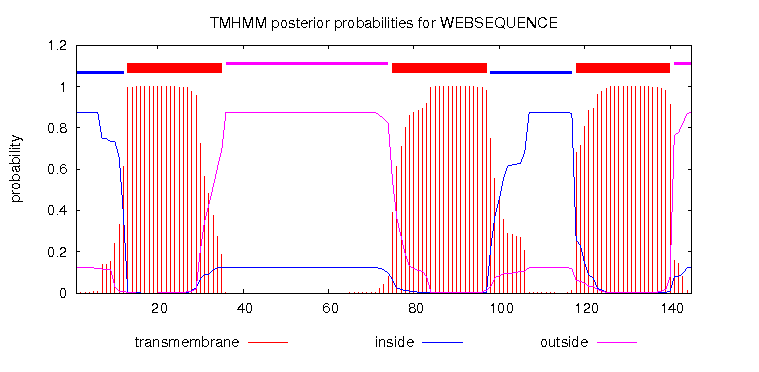

Supplement: Supplementary file 6 — Figure S4. The transmembrane region of CsMAPEG coding protein. (PNG 5 kb) [file 12870_2019_1971_MOESM6_ESM.png]

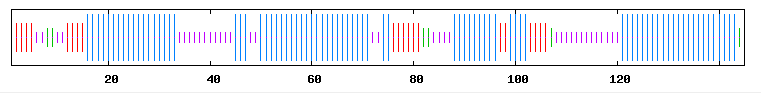

Supplement: Supplementary file 7 — Figure S5. Secondary protein structure of CsMAPEG. (PNG 1 kb) [file 12870_2019_1971_MOESM7_ESM.png]

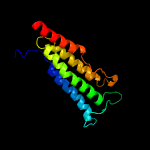

Supplement: Supplementary file 8 — Figure S6. Tertiary structure of protein. (PNG 9 kb) [file 12870_2019_1971_MOESM8_ESM.png]

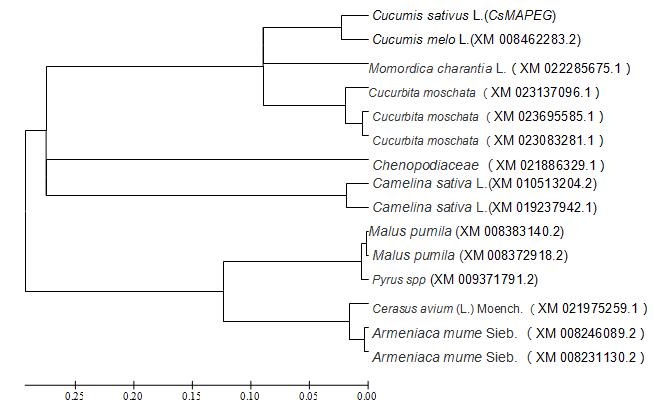

Supplement: Supplementary file 9 — Figure S7. Phylogenetic tree of CsMAPEG. The amino sequences were subjected to phylogenetic analysis using the neighbor-joining method in MEGA5.0 software, with 1000 bootstrap replicates. (JPG 35 kb) [file 12870_2019_1971_MOESM9_ESM.jpg]

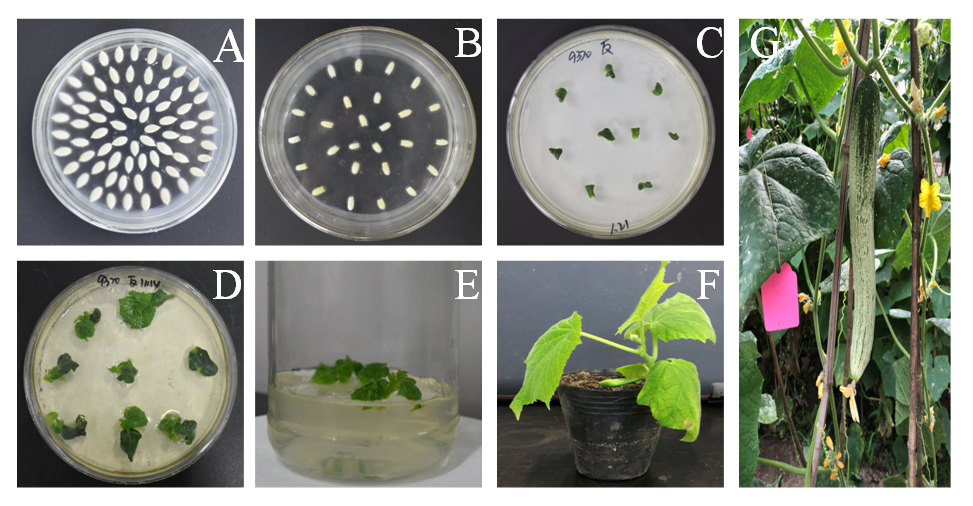

Supplement: Supplementary file 10 — Figure S8. pCXSN-CsMAPEG genetic transformation of cucumber. A:cucumber seed; B:co-culture; C:screening culture; D:plant regeneration; E:rooting culture of resistant seedlings; F:regeneration of resistant seedlings; G:seed of transgenic plants. (PNG 720 kb) [file 12870_2019_1971_MOESM10_ESM.png]
